# Single-nucleotide polymorphisms in dizygotic twin ovine fetuses are associated with discordant responses to antenatal steroid therapy

**DOI:** 10.1186/s12916-025-03910-9

**Published:** 2025-02-04

**Authors:** Erin L. Fee, Haruo Usuda, Sean W. D. Carter, Hideyuki Ikeda, Tsukasa Takahashi, Yuki Takahashi, Yusaku Kumagai, Michael W. Clarke, Demelza J. Ireland, John P. Newnham, Masatoshi Saito, Sebastian E. Illanes, Binny Priya Sesurajan, Liang Shen, Mahesh A. Choolani, Gokce Oguz, Adaikalavan Ramasamy, Sara Ritchie, Andrew Ritchie, Alan H. Jobe, Matthew W. Kemp

**Affiliations:** 1 Division of Obstetrics and Gynecology, Medical School, The University of Western Australia, Perth, WA Australia; 2 School of Biomedical Sciences, The University of Western Australia, Perth, WA Australia; 3 Centre for Perinatal and Neonatal Medicine, Tohoku University Hospital, Sendai, Japan; 4 Department of Obstetrics and Gynaecology, Yong Loo Lin School of Medicine, National University of Singapore, Singapore, Singapore; 5 Center for Microscopy, Characterization and Analysis, Metabolomics Australia, The University of Western Australia, Perth, WA Australia; 6 Department of Obstetrics and Gynecology, Faculty of Medicine, Universidad de los Andes, Santiago, Chile; 7 IMPACT, Center of Interventional Medicine for Precision and Advanced Cellular Therapy, Santiago, Chile; 8 Biostatistics Unit, Yong Loo Lin School of Medicine, National University of Singapore, Singapore, Singapore; 9 Genome Institute of Singapore. Agency for Science, Technology and Research (A*STAR), 60 Biopolis Street, Genome #02-01, Singapore, Singapore; 10 A8, Darkan, WA, Australia; 11 Cincinnati Children’s Hospital Medical Centre, University of Cincinnati School of Medicine, Cincinnati, OH USA; 12 School of Veterinary Medicine, Murdoch University, Perth, WA Australia; 13 Women and Children’s Hospital, Chongqing Medical University, Chongqing, China

**Keywords:** Betamethasone, Fetus, Fraternal twins, Glucocorticoids, Antenatal steroids, Sheep, Lung maturation, Preterm birth

## Abstract

**Background:**

Antenatal steroid (ANS) therapy is given to women at risk of preterm delivery to accelerate fetal lung maturation. However, the benefit of ANS therapy is variable and how maternal and fetal factors contribute to this observed variability is unknown. We aimed to test the degree of concordance in preterm lung function, and correlate this with genomic, transcriptomic, and pharmacokinetic variables in preterm dizygotic twin ovine fetuses.

**Methods:**

Thirty-one date-mated ewes carrying twin fetuses at 123 ± 1 days’ gestation received maternal intramuscular injections of either (i) 1 × 0.25 mg/kg betamethasone phosphate and acetate (CS1, *n* = 11 twin pairs) or (ii) 2 × 0.25 mg/kg betamethasone phosphate and acetate, 24 h apart (CS2, *n* = 10 twin pairs) or (iii) 2 × saline, 24 h apart (negative control, *n* = 10 twin pairs). Fetuses were surgically delivered 24 h after their final treatment and ventilated for 30 min.

**Results:**

ANS-exposed female fetuses had lower arterial partial pressure of carbon dioxide (PaCO_2_) values than male fetuses (76.5 ± 38.0 vs. 97.2 ± 42.5 mmHg), although the observed difference was not statistically significant (*p* = 0.1). Only 52% of ANS-treated twins were concordant for lung maturation responses. There was no difference in fetal lung tissue or plasma steroid concentrations within or between twin pairs. Genomic analysis identified 13 single-nucleotide polymorphisms (SNPs) statistically associated with ANS-responsiveness, including in the proto-oncogene *MET* and the transcription activator *STAT1.*

**Conclusions:**

Twin fetal responses and ANS tissue levels were comparable with those from singleton fetuses in earlier studies. Twin ovine fetuses thus benefit from ANS in a similar manner to singleton fetuses, and a larger dose of betamethasone is not required. Assuming no difference in input from the placental or maternal compartments, fetal lung responses to ANS therapy in dizygotic twin preterm lambs are dependent on the fetus itself. These data suggest a potential heritable role in determining ANS responsiveness.

**Supplementary Information:**

The online version contains supplementary material available at 10.1186/s12916-025-03910-9.

## Background

Preterm birth (being born earlier than 37 weeks’ gestation) is a leading cause of death in children under 5 years of age [1]. Being born preterm exposes babies to a greatly increased risk of neonatal mortality and morbidities (including respiratory distress syndrome (RDS), necrotizing enterocolitis (NEC), and intraventricular hemorrhage (IVH)), with risk proportional to the degree of prematurity [2–4].

Maternal administration of exogenous antenatal steroids (ANS; generally 24 mg of either dexamethasone phosphate or a combination of betamethasone acetate and phosphate) has been shown to reduce neonatal mortality and improve preterm outcomes by exerting maturational effects on fetal organs including the preterm lung and cardiovascular system [5]. When given to the right woman at the right time, ANS treatment prior to preterm delivery has the potential to save lives and reduce long-term disability. However, treatment response is variable, and a sizable amount of respiratory morbidity persists in ANS-treated preterm populations, demonstrating that a significant number of fetuses derive no benefit from ANS exposure [4, 6].

Previous studies have explored alternative ANS regimens, agents, doses, and target populations (including gestational ages and treatment to delivery intervals) to improve efficacy and further understand fetal responses to ANS [7]. Nevertheless, the cellular and molecular mechanisms through which ANS treatment induces lung maturation remain poorly understood, the issue of ANS response variability persists, and there is a paucity of evidence surrounding the administration of ANS for twin pregnancies (compared to singleton pregnancies), despite significantly higher rates of preterm birth and consequent ANS exposure for twin fetuses [8]. At present, most evidence based ANS guidelines are extrapolated from studies of singleton pregnancies only, highlighting the need for further investigation into ANS dosing and responsiveness in the setting of multiple pregnancy.

We have previously demonstrated that response variability may be influenced by ANS treatment protocol variations, including the drug used, the dose given, the treatment period, and the treatment to delivery interval [2, 9–11]. These treatment variables interact with individual fetal factors (including gestational age), along with maternal and/or placental inputs, to determine functional lung maturation responses. In the present study, we accounted for the above potential variability in ANS lung responses by using gestational age matched, dizygotic twin fetuses (i.e., receiving inputs from the same maternal compartment) exposed to a standardized ANS protocol for a fixed period. These animals were then surgically delivered and ventilated using a standardized protocol. Naturally occurring monozygotic twins are reported to account for less than 1% of all pregnancies in sheep [12]. Assuming equivalence between placental input, and by additionally measuring drug levels in the fetal plasma and lung tissue, the use of preterm dizygotic ovine twins allowed us to isolate any potential differences in lung responsiveness to the fetus itself. We thus used this controlled system to test our hypothesis that twin fetuses would have a strong degree of concordance in their lung maturation responses to standardized ANS therapy when undergoing postnatal ventilation.

## Methods

### Animal studies

We aimed to explore the degree of concordance in lung physiological and transcriptomic maturation responses to a standardized ANS protocol. Pregnant ewes were supplied by a single livestock provider and experiments were performed during the regular breeding season. Ewes were date-mated (24-h mating period, 1:5 ram to ewe ratio), and only ewes carrying twin fetuses were used. At 118 ± 1 days’ gestation, all ewes received an intramuscular (IM) injection of 150 mg medroxyprogesterone acetate (Depo-Ralovera®; Pfizer, West Ryde, NSW, Australia) to reduce the risk of preterm labor.

The study consisted of thirty-one ewes each carrying twin fetuses at 123 ± 1 days’ gestation. Group sizes were chosen to allow determination of differences of 2 standard deviation (SD) between treatment group means (steroid exposure vs. saline treatment) with power of at least 80% and a type-I error rate of 5%. We used previous, experimentally determined partial pressures of fetal arterial carbon dioxide (PaCO_2_) after 30 min of ventilation to determine group sizes using G*Power [13].

**Fig. 1:**
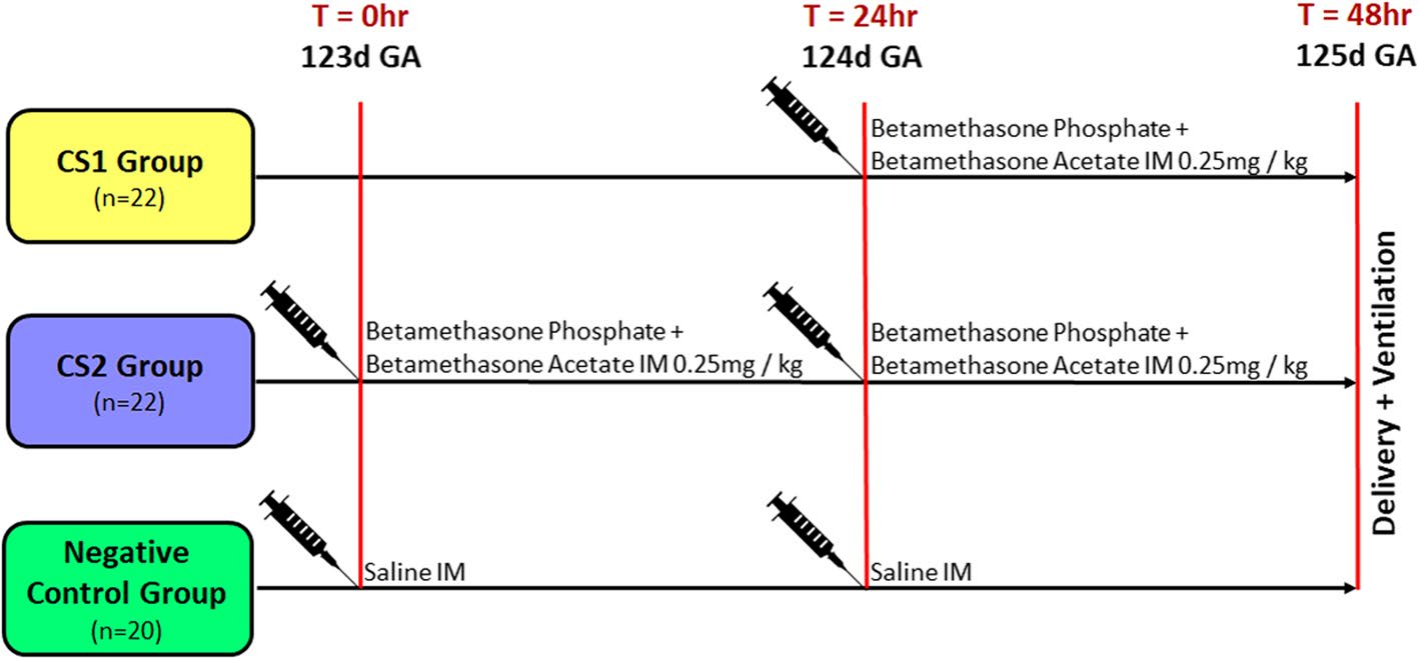
Schematic representation of experimental group structure showing interventions and group sizes (fetuses per group)

Animals were randomized to one of three groups (Fig. 1): Celestone 1 dose (CS1), group animals were given one maternal intramuscular (IM) injection of 0.25 mg/kg betamethasone phosphate + betamethasone acetate (Celestone® Chronodose®, Merck Sharp & Dohme, Australia) and delivered 24 h after this injection (*n* = 11 twin pairs/22 fetuses); Celestone 2 dose (CS2), group animals were given two IM injections of 0.25 mg/kg betamethasone phosphate + betamethasone acetate (Celestone® Chronodose®, Merck Sharp & Dohme, Australia) 24 h apart and delivered 48 h after the initial injection to replicate the current clinical regimen used across Australia and New Zealand (*n* = 10 twin pairs/20 fetuses); or negative control, group animals were given two IM injections of 2.0 ml saline 24 h apart and delivered 48 h after the initial injection (*n* = 10 twin pairs/20 fetuses). Ewes were weighed before intervention to calculate steroid dosing. Fetuses from all three groups were surgically delivered under terminal anesthesia at 125 ± 1 days’ gestation using a set protocol, ventilated for 30 min to allow lung function assessments, before being euthanized and subjected to necropsy [14]. No animals were used in other protocols.

### Preterm lamb ventilation

Prior to surgical delivery, pregnant ewes were anesthetized using an intravenous injection of midazolam (0.5 mg/kg) and ketamine (10 mg/kg), followed by a 3-mL spinal injection of 2% lidocaine (20 mg/mL). An abdominal incision was made, followed by hysterotomy allowing delivery of the head and neck of the lamb. Lambs then received an IM dose of ketamine (10 mg/kg) followed by tracheostomy and intubation using a 4.5-mm endotracheal tube, secured in place to create an airtight seal. The body of the lamb was then delivered, the umbilical cord clamped and cut before the lamb was dried, weighed and placed in a temperature controlled radiant warming bed (Cosy Cot, Fisher & Paykel Healthcare, New Zealand). Lambs were immediately ventilated for 30 min using Acutronic Fabian infant ventilators (Acutronic Medical System, Hirzel, Switzerland) delivering heated humidified oxygen (100%). The ventilation protocol used in this study was designed to allow standardized assessment of functional lung maturation. Ventilation parameters were standardized as follows: peak inspiratory pressure (PIP) of 35 cmH_2_O, positive end expiratory pressure (PEEP) of 5 cmH_2_O, respiratory rate of 50 breaths per minute, and an inspiratory time of 0.5 s. Tidal volume was controlled by adjusting peak inspiratory pressure to target a maximum tidal volume of 7–8 mL/kg. An umbilical artery catheter was placed to allow measurement of arterial blood for pH, partial pressure of oxygen (PaO_2_), PaCO_2_, heart rate, and blood pressure during ventilation. Ventilation data (compliance, tidal volume, PIP) were collected at 10-, 20-, and 30-min time increments. Ventilation efficacy index (VEI), an integrated measurement of ventilation, was calculated as follows: VEI = 3800/[respiratory rate (PIP – PEEP) × PaCO_2_ (mmHg)] [15]. The investigators who performed ventilations were blind to control or treatment group allocation.

### Defining ANS responsiveness

ANS responsiveness was defined using an arterial PaCO_2_ range developed using values from saline (null control) lambs after 30 min of ventilation. Previous studies using this model have shown that animals that fail to respond to ANS treatments have very poor lung maturation and are generally neither biologically nor statistically different from those exposed to placebo—based on PaCO_2_ values, as well as arterial pH, compliance and VEI [2, 10]. Accordingly, we have established a response range and threshold for antenatal steroid treatment responses in preterm lambs based on the normal distribution of data around a mean PaCO_2_ value for saline-treated animals. We set a threshold for determining antenatal steroid responsiveness as a 30-min PaCO_2_ value in any steroid-treated animal being more extreme than a value two standard deviations below that of the mean PaCO_2_ value in null-treatment (saline) animals (17). Above this cut-off, key markers of gas exchange (pH, lactate, BE) and ventilation (compliance, VEI) closely resemble animals treated only with saline. Thus, distinguishing between responders and non-responders in this manner provides a useful metric for assessing treatment success or failure.

### Necropsy

Ewes (after delivery) and lambs (after ventilation) were euthanized with intravenous injections of 160 mg/kg sodium pentobarbital. Lambs were weighed to record a post-ventilation weight. The lamb’s chest was then opened to visualize the lungs, and static lung compliance was measured by inflating the lungs from 0 to 40 cmH_2_O followed by controlled deflation to 0 cmH_2_O. The lungs were then removed and weighed separately. The right lower lobe was dissected and frozen for molecular and protein studies.

### Hematology and blood chemistry studies

Differential blood counts, liver function panels (aspartate aminotransferase (AST), alanine transaminase (ALT), gamma-glutamyl transferase (GGT), glutamate dehydrogenase (GLDH), bilirubin, urea, creatine, albumin, phosphate), and endocrine studies (cortisol, adrenocorticotropic hormone (ACTH), insulin-like growth factor 1 (IGF-1)) were performed by Vetpath Laboratory Services, Jandakot, Western Australia.

### Lung mRNA extraction for bulk sequencing and qPCR

Ribonucleic acid (RNA) was extracted from lung tissue (right lower lobe) using a RNeasy® Plus Mini Kit (QIAGEN, Hilden, Germany) as per the manufacturer’s instructions. The concentration of extracted RNA was determined using a broad-range acid quantitation kit (Life Technologies, Carlsbad, CA) and a Qubit 2.0 fluorometer (Life Technologies, Carlsbad, CA). RNA Integrity Number (RIN) was determined using Agilent Technologies RNA Nano Chip in accordance with manufacturer’s instructions. RNA extracts were diluted in nuclease-free water (Life Technologies) to achieve a final concentration of 25 ng/μL.

### Bulk RNA sequencing of preterm ovine lung tissue

A minimum RIN score of 7.5 was set for all samples submitted for sequencing. Libraries were prepared for directional bulk RNA sequencing (polyA enrichment) by Novogene Singapore using a NovaSeq X Plus (PE150) platform and sequenced to a depth of 30 million reads.

### Data processing for bulk RNA sequencing

150-bp paired-end sequenced reads were processed using the nf-core/rnaseq v3.10.1 pipeline [16] with nextflow v22.10.4 [17]. Briefly, raw reads were trimmed using Trim Galore! v0.6.7 [18] to remove low-quality bases and adapters. Trimmed reads were aligned to the Ovis aries genome assembly ARS-UI_Ramb_v2.0 with STAR v2.6.1d aligner [19]. Finally, Salmon v1.9.0 [20] was utilized for assigning reads. Principal component analysis (PCA) was conducted for quality control assessment and to identify data trends. All downstream analyses were performed using R statistical software version 4.2.1 [21]. Differential gene expression analysis was performed using the DESeq2 package v1.36.0 [22]. Genes with at least 1.5-fold change in expression and a false discovery rate (FDR) < 0.05 were considered significant. The results were visualized using the EnhancedVolcano v1.14.0 [23] and ComplexHeatmap v2.12.1 [24]. Gene set enrichment analysis required the full gene list. All genes were ranked by their signed log *p*-value for gene set enrichment analysis (GSEA) using clusterProfiler v4.9.0 [25] and Kyoto Encyclopedia of Genes and Genomes (KEGG) database [26].

### Measurement of transcript expression changes in the fetal lung using qPCR

Quantitative polymerase chain reaction (qPCR) cycling was performed with ovine-specific TAQMAN probe and primer sets (Applied Biosystems, Foster City, CA) using a Step One Real-Time PCR system in accordance with manufacturer’s instructions. Messenger RNA transcripts for surfactant protein A (*SFTPA*), surfactant protein B (*SFTPA*), surfactant protein C (*SFTPC*), and surfactant protein D (*SFTPD*). Amplification data for each gene was normalized to ribosomal protein *18 s* RNA. Delta quantification cycle values were used to determine relative expression of transcripts and for statistical analyses of between-group differences. Data is presented graphically as fold change compared to the negative control.

### Liquid chromatography-mass spectrometry (LC–MS)

Betamethasone levels in plasma and lung tissue (right lower lobe) were determined using liquid chromatography mass spectrometry (LC–MS) as reported previously [27]. Extraction of plasma samples and betamethasone standards (200, 100, 40, 20, 10, 2, 1, 0 ng/mL) were performed as follows: 50 μL of sample was added to 50 μL of internal standard (deuterated betamethasone, 50 ng/mL in 50:50 methanol: water + 0.1% formic acid) as described previously [27]. Samples were vortexed for 10 s and incubated at room temperature (RT) for 5 min. One milliliter of the solvent methyl tert-butyl ether (MTBE) was added; samples were sealed and vortexed for 2 min before being centrifuged at 3000 rpm for 10 min. Seven hundred microliters of clear sample was transferred to glass autosampler vials, dried under vacuum at 3000 rpm for 30 min at 37 °C, before being reconstituted in 70 μL in 50:50 methanol: water + 0.1% formic acid. Samples were capped and incubated with gentle shaking for 10 min at 50 °C, then analyzed.

Extraction of steroid from lung tissue used 100 mg of tissue weighed into a maceration tube. For standards, drug-free tissue from negative control animals was used with added plasma standard (0, 1, 2, 10, 20, 40, 100, 200 ng/mL). The extraction protocol was performed as follows: 50 μL of internal standard (deuterated betamethasone, 50 ng/ml in 50:50 methanol: water + 0.1% formic acid) [27] and 1 mL of the solvent methyl tert-butyl ether (MTBE) were added, and samples were macerated using a Precellys homogenizer at 6500 RPM. Samples were incubated for 1 h at 50 °C before being centrifuged at 3000 rpm for 20 min. Seven hundred microliters of clear sample was transferred to glass autosampler vials and treated following the remaining steps of the plasma protocol previously mentioned.

### Protein extraction and analysis

Protein was extracted from fetal lung tissue and analyzed for surfactant proteins according to previously published methods [14]. Protein in tissue protein extraction reagent (Life Technologies) buffer was used for measurement of surfactant proteins (SFTPA and SFTPB). A X-Cell SureLock Mini-Cell Electrophoresis System (Life Technologies) was used for electrophoresis and membrane transfer. Membranes were analyzed using an iBright FL100 Imaging System (Thermo Fisher). Target band concentrations were measured and normalized by total protein concentration. A standard quality control sample was used across all membranes to normalize values.

### Single-nucleotide polymorphism analysis

Single-nucleotide polymorphism (SNP) analysis of fetal genomic DNA was performed using the ovine-specific GeneSeek GenomicProfiler 50 k platform (51,817 individual SNPS; Neogen Bundamba, Australia). We filtered the SNP map to only include SNPs with a GenTrain Score above 0.7, ensuring high confidence in the genotyping process, and retained allele records with a GC Score over 0.15 to exclude low-quality genotype calls that could bias the results. SNPs with a *p* value of < 0.01 were included in downstream analyses. We then performed a logistic regression analysis to identify significant SNP combinations associated with ANS responsiveness, including interaction terms between SNPs and alleles, with sex as a covariate. Significant terms were filtered based on *p*-values (*p* < 0.05) and categorized by their direction of association. Additionally, we determined the zygosity of twin pairs by calculating concordance rates between their alleles, classifying pairs with concordance rates greater than 0.9 as monozygotic and others as dizygotic. All the SNP data analyses were performed using R version 4.3.2.

## Statistical analysis

Statistical analyses were performed using IBM SPSS Statistics for Windows, version 25.0 (IBM Corp, Armonk, NY). To compare continuous variables between groups (e.g., cord blood PaCO_2_ values, delivery weights, ventilation outcomes) Shapiro–Wilk tests were used to assess data normality. A Kruskal–Wallis test or one-way analysis of variance (ANOVA) was conducted according to data distribution (non-parametric or parametric data), followed by Tukey’s or Dunnett’s T3 post-hoc tests based on variance. A *p* value of < 0.05 was considered statistically significant. Significant differences between groups are expressed as (*p* value, mean difference, 95% confidence interval).

Chi-square test was used to assess the association between SNPs based on allele frequencies, identifying those with significant associations for further analysis. The cut-off value for this initial assessment was *p* < 0.01. These significant SNPs were then mapped to their respective gene names to provide biological context. Finally, we applied logistic regression for the shortlisted SNPs, adjusting for sex as a covariate to control for potential confounding factors, with a cut-off of *p* < 0.05.

A generalized estimation equation (GEE) model was used to evaluate the prognostic factors of treatment response of the correlated samples from twins, with CS1 and CS2 group animals pooled for analysis. Variables included in the model were as follows: treatment, fetal sex, twin type (Male + Male, Male + Female, Female + Female), and delivery order. An exchangeable structure as used for the correlation matrix. We used a cut-off of *p* < 0.05 and reported *p* value, odds ratio (OR), and the 95% confidence interval for the OR.

## Results

Thirty-one ewes and sixty-two lambs completed the protocol and are reported here.

For ventilation outcomes in ANS-exposed animals, lambs were identified as either ANS responders (having robust functional lung maturation) or ANS non-responders (having poor functional lung maturation) by comparing their PaCO_2_ values (an established measure of lung maturation) after 30 min of ventilation with ANS naïve animal (negative control) outcomes (Additional Fig. 1). As previously, we determined an experimentally derived cut-off value for ANS responsiveness using 30-min PaCO_2_ values from the negative control (i.e., steroid naïve) animals [10]. A PaCO_2_ cutoff value two standard deviations below the mean value of the of the negative control animal PaCO_2_ values was used. For this study, the PaCO_2_ cutoff was set at 84.3 mmHg. Accordingly, any ANS-treated animals with a 30-min PaCO_2_ value within two standard deviations of the negative control mean (i.e., higher than 84.3 mmHg) were classified as an ANS non-responder. Conversely, any ANS-treated animals with a 30-min PaCO_2_ value more extreme than two standard deviations of the negative control group mean (i.e., lower than 84.3 mmHg) were classified as an ANS responder.

### Delivery data

All fetuses were determined to be dizygotic, possessing SNP concordance rates of less than 0.9 (Additional Table 1) (concordance range 0.73–0.83).

All three study groups were analyzed for differences in delivery metrics, (gestational age, neonatal sex, birthweight, cord blood pH, cord blood PaCO_2_, lung weight normalized to post-ventilation body weight, white blood cell counts and blood chemistry), including responder and non-responder subgroups within the ANS-treated groups (determined post-delivery based on ventilation outcomes) (Table 1). Data were unremarkable. There were no statistically significant differences in birth weight, cord pH, or cord PaCO_2_ between steroid treatment group (CS1, CS2) and the negative control animals. There was a small, statistically significant (*p* < 0.001 95% CI − 12.3 to − 2.7 g) reduction in normalized wet lung weight between CS1 and the negative control animals. As anticipated, there were large, statistically significant (*p* < 0.001) perturbations in circulating fetal neutrophil and lymphocyte counts between steroid treatment group (CS1, CS2) and the negative control animals. Fetal plasma cortisol and ACTH were both significantly (*p* < 0.05) suppressed in the steroid-exposed group animals (Additional Table 2). Blood chemistry data were otherwise similar between groups (Table 1).

**Table 1:** Control and treatment group delivery data. There were no statistically significant differences in birthweight, cord pH or cord pCO_2_ between steroid treatment groups (CS1, CS2) and saline negative control animals. There was a small, statistically significant (*p* < 0.001) reduction in wet lung weight between CS1 and saline negative control animals. As anticipated, there were large, statistically significant (*p* < 0.001) perturbations in circulating fetal neutrophil and lymphocyte counts between steroid treatment groups (CS1, CS2) and saline negative control animals

Treatment group	CS1			CS2			Negative control
Sub-group	Total	Responders	Non-responders	Total	Responders	Non-responders	Total
*n*	22	12	10	20	12	8	20
Sex (male/female)	13/9	7/5	6/4	9/11	3/9	6/2	8/12
Gestation age (days)	125	125	125	125	125	125	125
Birthweight (kg)	2.97 ± 0.34	3.15 ± 0.27	2.76 ± 0.29	2.84 ± 0.42	2.86 ± 0.37	2.81 ± 0.52	2.71 ± 0.38
Cord blood pH	7.28 ± 0.11	7.25 ± 0.13	7.32 ± 0.05	7.29 ± 0.14	7.32 ± 0.12	7.26 ± 0.18	7.22 ± 0.10
Cord blood PaCO_2_ (mmHg)	60.68 ± 13.59	63.44 ± 16.11	56.99 ± 8.84	59.05 ± 16.10	57.36 ± 11.03	61.38 ± 21.96	66.15 ± 11.14
Lung weight (g/kg)	30.95 ± 5.22*	30.15 ± 5.15*	31.90 ± 5.42*	34.24 ± 4.93	33.24 ± 4.38	35.73 ± 5.62	38.47 ± 5.51
Cord blood values							
Total WBC (× 10^9^/L)	4.4 ± 1.15	4.48 ± 0.81	4.39 ± 1.51	3.57 ± 0.65	3.39 ± 0.60	3.90 ± 0.66	4.17 ± 1.29
Neutrophils (× 10^9^/L)	2.53 ± 1.16*	2.52 ± 0.93*	2.54 ± 1.44*	1.91 ± 0.70*	1.63 ± 0.65*	2.41 ± 0.52*	0.34 ± 0.27
Lymphocytes (× 10^9^/L)	1.65 ± 0.60*	1.68 ± 0.62*	1.63 ± 0.61*	1.60 ± 0.65*	1.69 ± 0.73*	1.43 ± 0.47*	3.48 ± 1.04

Values are expressed as mean ± one standard deviation

^*^p < 0.001 vs negative control group

### Betamethasone measurements

LC–MS was used to measure betamethasone levels in fetal and maternal plasma samples collected at delivery and fetal lung samples collected at necropsy. The limit of detection in plasma was defined as a betamethasone concentration of 0.5 ng/mL with a signal to noise ratio of < 10:1. The limit of detection in lung tissue was defined as a betamethasone concentration of 1 ng/ml with a signal to noise ratio of < 10:1.

Maternal and fetal plasma betamethasone concentrations at delivery are reported in Table 2. The absolute difference in fetal plasma betamethasone concentration for twin sets were calculated. The average absolute difference in fetal plasma concentrations was 0.12 ng/mL and 0.22 ng/mL in the CS1 and CS2 groups respectively. No significant differences in fetal plasma betamethasone concentrations were identified between male vs. female (CS1 *p* = 0.74, CS2 *p* = 0.26) or ANS responder vs. non-responder (CS1 *p* = 0.58, CS2 *p* = 0.60) animals for either ANS treatment group.

**Table 2:** Plasma and lung tissue betamethasone concentration at delivery. No significant differences in fetal plasma betamethasone concentrations were identified between male vs. female (CS1 *p* = 0.74, CS2 *p* = 0.26) or ANS responder vs. non-responder (CS1 *p* = 0.58, CS2 *p* = 0.60) animals within either ANS treatment group

		**Mean**	**SD**	**Mean absolute difference between twins**
**Maternal plasma**				
**CS1**		14.8	11.4	-
**CS2**		7.8	2.2	-
**Negative control group**		< 1	-	-
**Fetal plasma**				
**CS1**	Total (*n* = 22)	2.5	1.5	0.22
	Male	2.5	1.5	-
	Female	2.5	1.5	-
	Responder	2.4	1.3	-
	Non-responder	2.7	1.7	-
**CS2**	Total (*n* = 20)	1.6	0.3	0.12
	Male	1.7	0.4	-
	Female	1.6	0.2	-
	Responder	1.7	0.3	-
	Non-responder	1.6	0.3	-
**Fetal lung tissue**				
**CS1**	Total	42.6	19.4	6.9
	Male	43.7	21.7	-
	Female	41.0	16.6	-
	Responder	40.1	15.2	-
	Non-responder	45.6	24.0	-
**CS2**	Total	20.7	4.9	1.8
	Male	23.0	4.5	-
	Female	18.9	4.6	-
	Responder	21.1	4.3	-
	Non-responder	20.1	5.9	-

Fetal lung tissue betamethasone concentrations are reported in Table 2. Absolute differences in tissue betamethasone concentrations for twin pairs were calculated. The average absolute difference in fetal lung tissue betamethasone concentration was 6.9 ng/mL and 1.8 ng/mL in the CS1 and CS2 groups respectively. No significant differences in fetal lung tissue betamethasone concentration were identified between male vs. female (CS1 *p* = 0.85, CS2 *p* = 0.06) or ANS responder vs. non-responder (CS1 *p* = 0.67, CS2 *p* = 0.67) animals for either steroid treatment group. As expected, betamethasone was not detected in fetal and maternal samples collected from the negative control animals.

### Ventilation data (30 min)

Fetuses delivered first were less likely to be ANS responders than fetuses delivered second (adjusted OR 0.258; *p* = 0.031, 95% CI 0.076–0.880). Delivery of both fetuses was completed in less than 3 min. There was no difference in cord blood gas measurements (i.e., PaCO_2_, pH, PaO_2_, lactate) between first and second delivered fetuses. The delivery order effect was not impacted by fetal sex.

Mean PaCO_2_ values and respiratory response rate for ANS-treated groups are reported in Table 3. The mean 30-min PaCO_2_ value for negative control (steroid naïve) lambs was 130.7 ± 28.3 mmHg for females and 129.1 ± 19.5 mmHg for males, and these were not significantly different (*p* = 0.88). For ANS-treated animals, mean PaCO_2_ values for female (F) and male (M) fetuses were 76.5 ± 38.0 mmHg and 97.2 ± 42.5 mmHg, respectively, and these were not significantly different (*p* = 0.10). Fifty-two percent of all twin pairs were concordant for ANS lung responses (i.e., both twins were either ANS responders or non-responders). Female fetuses (5/9 in the CS1 group and 9/11 in the CS2 group) were more likely to respond to ANS treatment than males (7/13 in the CS1 group and 3/9 in the CS2 group) (adjusted OR 7.786; *p* = 0.018, 95% CI 1.42—42.680).

**Table 3:** ANS treatment response rate. The mean 30-min PaCO_2_ values for male (M) and female (F) negative control lambs were not significantly different (*p* = 0.88). For ANS-treated animals, mean PaCO_2_ values for female and male fetuses were not significantly different (*p* = 0.10). Fetuses delivered first were less likely to be ANS responders than fetuses delivered second (OR 0.258; *p* = 0.031, 95% CI 0.076 – 0.880). Female fetuses (5/9 in the CS1 group and 9/11 in the CS2 group) were more likely to respond to ANS treatment than males (7/13 in the CS1 group and 3/9 in the CS2 group) (OR 7.786; *p* = 0.018, 95% CI 1.42—42.680)

	**Sex**	**PaCO**_**2**_** mean (mmHg)**		**SD**	**Response rate**	**%**
**All (*n* = 42)**						
**CS1**	Male		85.5	27.6	7 (13)	54
	Female		88.6	49.9	5 (9)	56
**CS2**	Male		114.1	55.3	3 (9)	33
	Female		66.6	22.6	9 (11)	82
**Male/female twin pairs (*n* = 28)**						
**CS1**	Male		91.39	29.9	4/9	44.4
	Female		82.19	52.0	5/9	56
**CS2**	Male		126.7	53.1	1/5	20
	Female		61.1	12.3	5/5	100
**Male/male twin pairs (*n* = 8)**						
**CS1**	-		72.4	23.9	3/4	75
**CS2**	-		98.3	61.5	2/4	50
**Female/female twin pairs (*n* = 6)**						
**CS1**	-		-	-	0/0	-
**CS2**	-		71.2	29.01	4/6	67

PaCO_2_ cutoff is used to define responder vs non responder. PaCO_2_ cutoff is calculated as 2 STD below the average of the control group = 84.3

Negative control animals (treated with saline) are not included in this analysis

### Bulk RNA sequencing

**Fig. 2:**
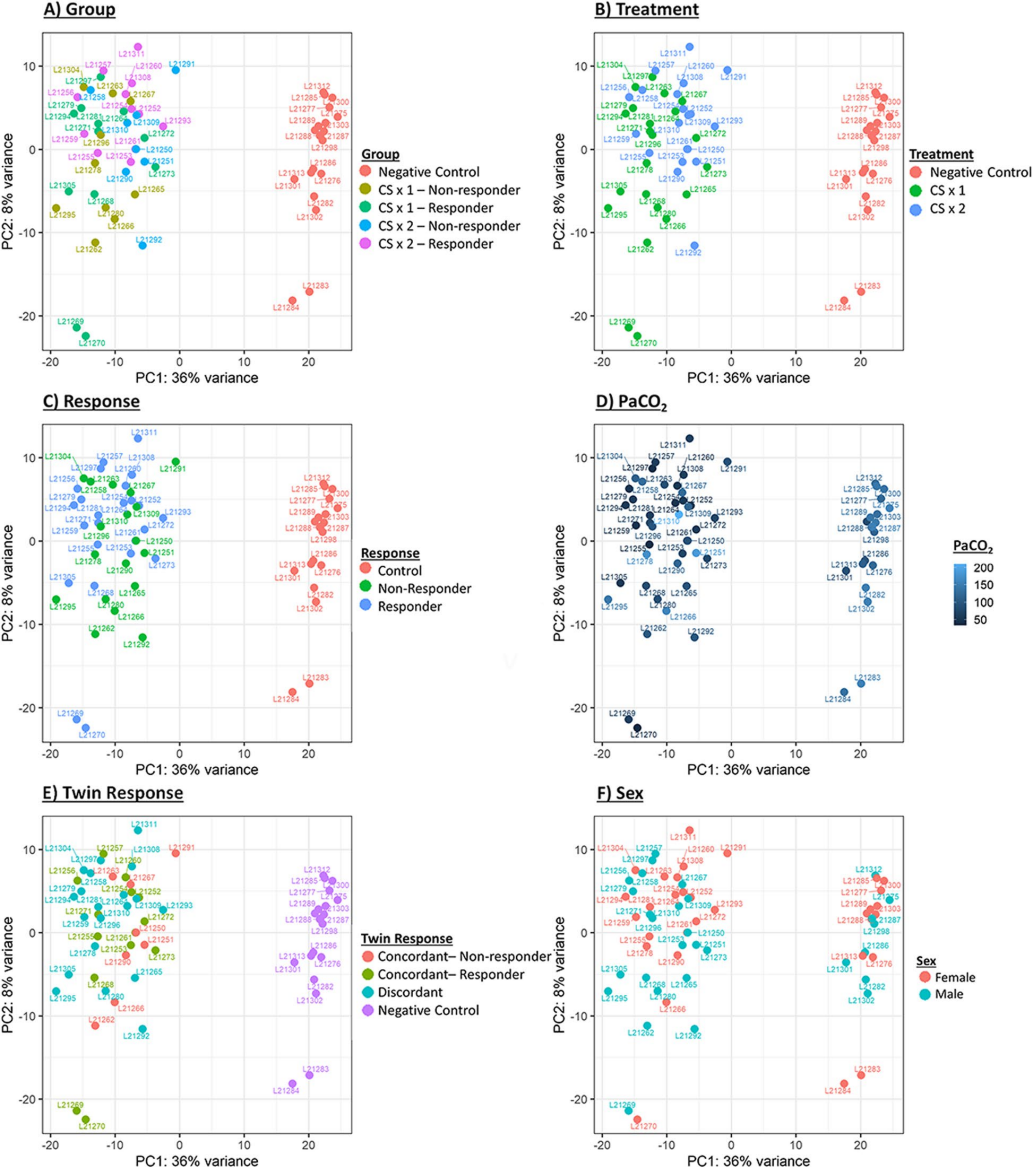
PCA plots of bulk RNA sequencing data from the lower right lobe of the preterm fetal lung

Lung RNA from all sixty-two lambs was submitted for bulk sequencing analysis and quantification of mRNA levels. PCA plots revealed distinct clustering of the negative control animals and steroid-treated animals (Fig. 2). No distinct clustering was observed for animals by steroid exposure group (i.e., CS1 vs. CS2), ANS response status (i.e., ANS responder or non-responder), fetal sex, or twin ANS response concordance.

When adjusted for treatment and sex, 16, 386 known transcripts were identified, and twenty-three genes were determined to be significantly different between ANS responder (*n* = 24) and non-responders (*n* = 18); there were nine upregulated and fourteen downregulated (Additional Table 3). A heatmap was used to identify difference between the negative control and ANS-treated groups and responder/non responder subgroups for genes associated with lung maturation, increased vasculature, inflammation/immune regulation, extracellular matrix (ECM)/cellular matrix, and cell division/cycle (Additional Fig. 2).

### Quantitative polymerase chain reaction (qPCR) analysis of transcript expression changes in the fetal lung

qPCR of surfactant proteins A-D was used to identify expression difference between responder and non-responders. There was no significant difference in the expression of surfactant proteins between responders and non-responders (Additional Fig. 3), reflecting the bulk sequencing results.

### Western blot analysis of lung tissue

There was a small, statistically significant increase in the normalized protein band volume for SFTPA in the CS2 vs. negative control animals (0.97 ± 0.33 vs. 0.68 ± 0.31; *p* = 0.003). Among the CS2 animals, there was no difference in normalized protein band volume for SFTPA between ANS-responder and ANS-non responder animals. There was no difference for SFTPB band volumes. CS1 animals were not studied.

### Single-nucleotide polymorphism analyses

Thirteen SNPs identified as being significantly associated with ANS treatment responsiveness are shown in Table 4, along with identified allele forms. Of these, SNPs in insulin-like growth factor binding protein 6 (*IGFBP-6*) were uniquely associated with ANS non-responsiveness, and SNPs in eyes absent transcriptional coactivator and phosphatase 3 (*EYA3*), indoleamine 2,3-dioxygenase 1 (*IDO1*), MET proto-oncogene, receptor tyrosine kinase (*MET*), stearoyl-CoA desaturase 5 (*SCD5*), and signal transducer and activator of transcription 1 (*STAT1*) were uniquely associated with ANS responsiveness. SNPs in APAF1-interacting protein gene (*APIP*), calpain 3 (*CAPN3*), CD53 molecule (*CD53*), leptin receptor (*LEPR*), nuclear receptor subfamily 3 group C member 1 or glucocorticoid receptor (*NR3C1*), and shroom family member 4 (*SHROOM4*) were statistically associated with both ANS responsiveness and non-responsiveness.

**Table 4:** Fetal single-nucleotide polymorphism (SNPs) statistically associated with ANS responsiveness

SNP	Gene	Chromosome	Position	Effect	Reference allele	Responder allele	*p*-value Cut-off ChiSQ *p*-value < 0.01 LR *p*-value < 0.05	Non-responder allele	*p*-value Cut-off ChiSQ *p*-value < 0.01 LR *p*-value < 0.05
OAR15_69180648.1	APIP	15	63,627,667	Intron variant	C	Allele1 – Forward C***	0.001 0.0009	Allele2 – Forward C*	0.008 0.030
oar3_OAR7_34804896	CAPN3	7	34,804,896	3′ UTR variant	C	Allele2 – Forward C***	0.0003 < 0.0001	Allele1 – Forward C*	0.006 0.029
oar3_OAR1_87387610	CD53	1	87,387,610	Intron variant	A	Allele2 – Forward A***	0.001 < 0.0001	Allele1 – Forward A*	0.008 0.038
OAR2_251639875.1	EYA3	2	238,259,883	Intron variant	A	Allele1 – Forward A**	0.003 0.006	-	
oar3_OAR3_133208610	IGFBP6	3	133,208,610	Downstream gene variant	T	-		Allele1 – Forward C*	0.006 0.034
oar3_OAR1_40819378	LEPR	1	40,819,378	Intron variant	A	Allele1 – Forward A**	0.007 0.008	Allele1 – Forward G***	0.0002 < 0.0001
oar3_OAR4_51540896	MET	4	51,540,896	Intron variant	A	Allele2 – Forward A*	0.005 0.019	-	
s64770.1	MET	4	51,565,726	Intron variant	C	Allele1 – Forward C*	0.005 0.019	-	
OAR5_56257273.1	NR3C1	5	51,843,084	Intron variant	T	Allele2 – Forward C*	0.009 0.021	Allele1 – Forward C***	0.001 < 0.0001
oar3_OAR6_97080753	SCD5	6	97,080,753	Intron variant	T	Allele1 – Forward C*	0.005 0.016	-	
zoar3_OARX_51195419	SHROOM4	X	51,195,419	Intron variant	T	Allele2 – Forward C***	0.0006 0.0008	Allele1 – Forward C**	0.009 0.007
oar3_OAR2_192174047	STAT1	2	192,174,047	Intron variant	T	Allele1 – Forward C*	0.002 0.019	-	

*LR* Logistic regression, *ChiSQ* Chi-square test

*p < 0.05

**p < 0.01

***p < 0.001

## Discussion

### Principal findings

The principal findings of this study were as follows:

i) That fetal respiratory responses to ANS treatment in dizygotic preterm lambs were dependent on the fetus, but not maternal inputs;
ii) Variability in responsiveness to ANS treatment was not impacted by the regimen delivered (i.e., a half (CS1 group) or complete (CS2 group) course of betamethasone phosphate and acetate), or associated with differences in delivery characteristics, delivery order or fetal tissue betamethasone levels;
iii) Steroid naïve male and female fetuses had equivalent ventilation outcomes. When exposed to ANS, male fetuses had higher PaCO_2_ ventilation values and were more likely to be classified as having functionally immature lungs (ANS non-responsive). When ANS-responsive, male fetuses had broadly equivalent ANS responses (when comparing ventilation outcomes, e.g., improved lung compliance, VEI values, gas exchange) to females;
iv) Bulk sequencing of lung RNA showed little significance in gene expression, in particular no significant difference was identified in genes affiliated with lung maturation between the responders and non-responders;
v) Genomic analysis identified 13 SNPs associated with 12 genes, including one position in the glucocorticoid receptor, *NR3C1*, that were statistically associated with changes in ANS responsiveness. Twelve SNPs in 11 genes were probabilistically associated with ANS responsiveness, whereas 7 SNPs in 7 genes were associated with ANS non-responsiveness. Within this SNP set, one SNP in *IGFBP-6* was uniquely associated with non-responsiveness, and SNPs in *EYA3*, *IDO1*, *MET*, *SCD5*, and *STAT1* were uniquely associated with ANS responsiveness.
vi) Taken as a whole, these data suggest, assuming appropriate drug exposure (i.e., dose and duration of glucocorticoid exposure relative to delivery timing), that fetal genetics (i.e., fetal sex and SNP patterns) play a key role in dictating the functional lung responses of ovine fetuses to ANS treatment. Given the strong evolutionary conservation of several of the genes identified in the SNP analysis (notably *NR3C1* and *STAT1*), these data warrant functional studies on the variants identified in this study, in conjunction with human SNP studies in the setting of residual respiratory morbidity following appropriately targeted ANS treatments.

### Results in the context of what is already known

Sizable uncertainty remains regarding the optimal use of ANS in twin pregnancies. Socha and colleagues recently reported a meta-analysis concluding that data from non-randomized studies across a wide gestational age range (23–36 weeks’ gestational age) suggested a benefit in terms of neonatal death and respiratory disease risk. These conclusions are in keeping with work by Melamed [28] (Canadian preterm twin population 24^0/7^ to 33^6/7^ weeks’ gestation, ANS therapy was associated with a clinically significant reduction in death and acute respiratory morbidity), Vaz [29] (Portuguese preterm twin population 25^0/7^ to 34^6/7^ weeks’ gestation, ANS were associated with improvement in neonatal outcomes for both singletons and twins), and Palas [30] (French Epipage-2 cohort, 24^0/7^ to 31^6/7^ weeks’ gestation, ANS associated with reduced rate of periventricular leukomalacia or intraventricular hemorrhage). In contrast, benefit data in the late-preterm period is less clear. Retrospective analyses from Israel [31] (Ben-David et al., twin preterm population 34^0/7^–36^6/7^ weeks’ gestation, ANS were associated with no risk of respiratory disease but increased risk of hypoglycemia) and China [32] (Zhu et al., twin preterm population 34^0/7^–36^6/7^ weeks’ gestation, insufficient evidence that ANS was associated with lower morbidity) do not support the use of ANS in this population. The ACTWIN study from South Korea remains unreported [33] and the STOPPIT-3 Trial in the United Kingdom is ongoing [34]. Although most directly relevant to preterm infants (125d gestation in the sheep approximates lung development around 32 weeks’ human gestation) [35], our data add support for ANS conveying similar respiratory benefit to twin fetuses as seen in singleton fetuses.

Our data further emphasize the previously reported relationship between fetal sex and ANS responsiveness, with female fetuses more likely to respond to steroid treatment. Variability in ANS response as a function of fetal sex is incompletely understood and data is contradictory. Previous studies in the sheep model of pregnancy concluded that female preterm lambs had marginally better, but non-statistically significant improvements in lung responses to betamethasone [36]. Human data are somewhat unclear with regards to the effect of fetal sex on ANS treatment efficacy. Ballard and colleagues reported, in a small cohort of ANS-exposed preterm infants (1251 to 1750 g in birth weight), that the incidence of respiratory distress syndrome (RDS) was 40.9% in males and 7.1% in females (*n* = 22 and 14, respectively; *p* = 0.03) and concluded reduced efficacy in males [37]. In analyzing respiratory outcomes of a cohort of lower birth weight babies (710 patients; birth weight IQR 800–1391 g), Ramos-Navarro et al. reported a positive benefit from ANS treatment in males with a gestational age of less than 29 weeks [38]. Similarly, the findings from the recent update to the Auckland Steroid Study reported a greater effect in male than in female infants [39]. A 2010 meta-analysis of the literature (not including the updated Auckland data) based on 1109 male and 968 female infants concluded that both male and female preterm infants had an equivalent reduction in risk of RDS after ANS therapy but did suggest potential outcome differences based on the glucocorticoid regimen employed [40]. In the present study, we report no sex differences in steroid-naïve lung function, but a significant association between steroid responsiveness and fetal sex, favoring females. Interestingly, for those fetuses classified as ANS responders, despite a clear trend favoring females, no significant difference in PaCO_2_ values between steroid-treated male and female fetuses was observed. This could be explained by the relatively small number of individuals in the study or that although male sheep fetuses are less likely to respond to ANS therapy, those that do gain equivalent benefit to female fetuses.

### Clinical implications

The key clinical implications of these data include (i) providing further support for the use of ANS in twin pregnancies and (ii) that administration of higher doses of ANS are not required to have a beneficial effect in twin pregnancies. In the present study, administration of either a half (CS1 group) or complete (CS2 group) course of betamethasone phosphate and acetate was associated with statistically significant and biologically important improvements in preterm lung maturation, demonstrated by improved gas exchange, better static (at necropsy) and dynamic (under ventilation) compliance, and improved VEI values. An assessment of our previous work in this model [10] showed that the magnitude of benefit conveyed to twin fetuses by ANS was not significantly different to age-matched singleton fetuses. These data thus support the judicious use of ANS in twin fetuses in the same manner as for singleton fetuses.

Using sheep and non-human primate models, we and others have shown that once a minimum concentration of fetal glucocorticoid exposure has been achieved and maintained uninterrupted for a sufficient period, variability in preterm lung maturation is not a function of variability in fetal plasma or tissue drug levels [6, 11]. To date, these studies have only been conducted in singleton pregnancies. The present data extend these findings to twin ovine fetuses, wherein there was negligible difference in fetal drug levels between twin pairs, regardless of fetal sex or whether a half (CS1) or complete (CS2) course of betamethasone acetate and phosphate was administered. As previously discussed, the very long half-life of the betamethasone acetate component of this treatment provides sufficient magnitude and duration of fetal betamethasone exposure to materially improve lung maturation, relative to control, at a 48-h treatment to delivery interval [7]. An additional implication of these data (noting a higher materno-fetal glucocorticoid transfer in the human) is that current ANS dosing is likely also excessive for twin pregnancies, as in the case with singleton pregnancies.

Comparing our data with earlier singleton pregnancy studies, there was little difference in fetal drug levels in the context of a multiple pregnancy. In a chronically catheterized model receiving a single 0.25 mg/kg maternal intramuscular injection of betamethasone phosphate and acetate, maternal and fetal plasma betamethasone levels were 11.9 ± 3.9 ng/mL and 1.9 ± 0.55 ng/mL after 24 h, neither of which were significantly (*p* = 1.0 for both comparisons) different from the twin maternal and fetal values reported in the present study [10]. These findings provide reassurance that twin pregnancies do not require higher maternal dosing to achieve efficacious levels of fetal drug exposure. These findings in sheep are also consistent with human data (i.e., multiple pregnancies do not alter betamethasone pharmacokinetic parameters) reported previously by Della Torre and colleagues [41]. In contrast, Ballabh and colleagues have reported a small difference in betamethasone half-life in twins compared with singletons (7.2 ± 2.4 h vs. 9.0 ± 2.7 h; *p* < 0 0.017) [42], which likely carries minimal clinical significance. Taken together, these data suggest that there is little or no difference in fetal betamethasone exposures between singleton and twin pregnancies. This is an important consideration given that twin pregnancies are at higher risk of preterm delivery than singleton pregnancies [43], increasing the likelihood of twin preterm infants being admitted to higher support neonatal care units [44]. Similarly, our data support earlier data that the materno-fetal pharmacokinetics of betamethasone in the pregnant sheep are not materially different between singleton and twin fetuses. Accordingly, these data argue against the need for a higher ANS dosing regimen in twin pregnancies.

### Research implications

By standardizing pre-ANS exposures (i.e., housing, feed, environment, stress), fetal gestational age, the ANS regimen, the mode of delivery, and the post-natal ventilation parameters, we were able to use dizygotic twin fetuses to isolate any potential differences in ANS-responsiveness to the level of the individual fetus. Our hypothesis that twin sheep fetuses would show statistically and biologically significant concordance in ANS-induced preterm lung maturation is rejected following analysis of the ventilation data, showing only 52% concordance in ANS-response between twin fetuses.

The exact mechanism by which ANS exposure drives fetal lung maturation, and thus the basis of the observed response variability, remains unclear. However, our data support the conclusion that the maternal compartment does not play an important role in this process. In focusing on the role of the fetal compartment in this process, it is important to note that we are unable to isolate a potential contribution from the fetal placenta, as opposed to the fetus alone. That said, our previous work showing robust lung maturation following low-concentration steroid infusions directly to the fetus (therefore bypassing the placenta) add weight to our view that, adequate growth support aside, ANS-driven lung maturation is independent of placental input.

Of particular interest from a research perspective are our data relating to (i) the sizable degree of discordance between dizygotic twins to ANS treatment and the association of ANS responsiveness with fetal SNP patterns, (ii) the effect of fetal sex on ANS responsiveness, and (iii) the similarity in lung gene expression between animals that responded and did not respond to ANS exposures.

In the present study, a lack of mechanistic data limits our ability to understand the functional implication of the observed SNPs on ANS-response potential. Although several investigators have studied SNP associations with altered glucocorticoid sensitivity across multiple disease phenotypes, comparatively little attention has been paid to how genetic variants might impact fetal responses to ANS therapy. In the present study, we identified an intronic variant (T/C) in *NR3C1*, with a variant in allele 2 associated with steroid responsiveness and an equivalent variant in allele 1 associated with non-responsiveness. Accordingly, the functional implications of this SNP association are unclear. Van Rossum and colleagues identified rs41423247, otherwise known as the *BclII* restriction site polymorphism, a C/G substitution in *NR3C1* exon 2 in a Dutch population cohort [45]. Heterozygous (CG) and homozygous (GG) allele carriers were associated with glucocorticoid hypersensitivity. Furthermore, Haas and colleagues reported maternal rs41423247 to be associated with neonatal respiratory disease in the setting of ANS therapy [46]. An additional polymorphism ER22/23EK, also in exon 2 of *NR3C1* was associated with bronchial asthma and glucocorticoid resistance in a Polish population [47].

In the *NR3C1* gene of sheep, the SNP OAR5_56257273.1 at position 5: 51,843,084 with the mutation T—> C at the genomic level, A—> G at the transcript level, and resulting in an M—> V (methionine to valine) substitution at the protein level, is associated with both drug responsiveness and non-responsiveness. The M to V substitution may alter the *NR3C1* protein's structure and binding affinity to glucocorticoid drugs, with varying effects depending on the genetic and environmental context, thus influencing the treatment outcomes.

One SNP (*IGFBP6*) was found exclusively in association with ANS non-responsiveness. *IGFBP6* is a member of the insulin-like growth factor binding protein family, with both complex insulin-like growth factors modifying their activity [48]. Recent data suggest a role for these proteins in tissue remodeling, repair, and fibrosis, and IGFBP6 has been shown to be expressed in the developing fetal rat lung [49]. SNPs in the 5′ terminal IGFBP6 sequence have been linked to body size regulation in pigs [50].

Six SNPs (*EYA3*,* IDO1*,* SCD5*,* MET*,* STAT1*) were found exclusively in association with ANS-responsiveness. *EYA3* has been shown to have tyrosine phosphatase activity, playing a role in the development and maintenance of vascular remodeling [51]. Wang et al. concluded that the EYA’s protein tyrosine phosphatase activity promoted survival of lung vascular cells in the setting of DNA damage [51]. *IDO1* is an enzyme in the kynurenine pathway that catalyzes the degradation of tryptophan [52]. It plays a variety of immunoregulatory and signaling roles, including β-catenin signaling (noting the importance of WNT/β-catenin signaling in fetal lung development [53, 54]). It is also reported to be involved in maintaining embryonic stem cell pluripotency and is downregulated with the onset of differentiation [52]. *SCD5* is a stearoyl-CoA desaturase involved in monounsaturated fatty acid synthesis. Its biological importance to the developing fetal lung is uncertain, although SNPs in the *SCD5* promoter region have been linked to diabetes mellitus [55].

More interesting from a fetal lung maturation perspective are SNPs identified in *MET* and *STAT1*. *MET* encodes a receptor tyrosine kinase c-MET that is expressed in epithelial cells acts as a receptor for mesenchymal hepatocyte growth factor (HGF) [56]. HGF has been shown to play an important (but not essential) role in branching morphogenesis in the fetal rat lung and is believed to exert a synergistic effect with keratinocyte growth factor [57]. Increases in HGF expression are also seen in association with acute lung injury [58]. SNPs in *MET* have been associated with a range of cancers, including of the lung [59]. Lastly, the signal transducer and activator of transcription (*STAT*1) family of proteins are transcription factors that modulate signaling from a range of growth factors and cytokines. Data from mice suggest that STATs are not essential for morphogenesis but do play a role in lung inflammation and repair [60]. *STAT1* plays an important role in type I and II interferon signaling, with knockout mice susceptible to infection [61]. Xu and colleagues identified *STAT1* as a key transcription factor node in day 18.5 embryonic mouse lung, noting that Janus kinase/STAT signaling plays important roles in cell growth, apoptosis, and differentiation [62]. STAT proteins are also important in directing endothelial growth factor receptor signaling [62], with insufficient endothelial growth factor expression linked to respiratory distress syndrome [63].

Although a secondary finding, the impact of fetal sex on ANS-responsiveness is of interest. Sex effects on singleton pregnancies have been addressed above. Although a comparatively small number of retrospective studies (also discussed above) have focused on twin responses to ANS therapy, there has been limited focus on the treatment outcomes of individual fetuses in twin pairs or how those outcomes were associated with fetal sex. Given the likely availability of these data, this should be considered a research priority, especially with regards to preterm respiratory disease and long-term follow-up. In a small sequential ultrasound study of twin pregnancies, Mulder and colleagues concluded that there was a high degree of concordance in betamethasone-induced fetal effects (fetal heart rate variability, body and breathing movements) between twin pairs, with no effect of sex, position, or size [64]. The authors also concluded that the observed betamethasone effects were the same in twin and singleton fetuses. The effect of twin sex combinations on lung maturation has also not been well explored; there are some data available to suggest a bi-directional effect on growth and development. Luke and colleagues provide an extensive summary of twin sex effects, with mixed twin pairs reported to have differential effects (relative to matched sex twins) on birth weight [65]. A historical study by Lummaa and colleagues reported that females born with a male twin had reduced lifetime reproductive success compared to those born with a female twin [66]. A number of investigators have similarly hypothesized that transfer of androgens or estrogens between fetuses, or differential sensitivities to these mediators, exerts a range of post-natal effects, notably including masculinizing effects on females resulting in increased attention-seeking behavior and aggression [65]. Whether inter-fetal transfer of hormones can similarly contribute to altered lung maturation remains unsubstantiated but is worthy of additional study.

Lastly, the lack of differences in the fetal lung transcriptome (23 differentially regulated genes, each with quite modest changes in terms of magnitude and statistical significance) between ANS responders and non-responders identified herein was somewhat surprising and inconsistent with the sizable physiological differences seen between these two groups on animals. Lung tissues were sampled from a set lower marginal area of the right lower lobe, before being snap frozen, with RNA sequenced to a depth of 30 million reads. It is possible that the technique employed (i.e., bulk RNA sequencing as opposed to single-nucleus sequencing) and/or the read depth used were insufficiently sensitive to detect subtle transcriptomic changes, especially if transcriptional changes were restricted to a small subset of interacting epithelial and mesenchymal cells [67]. It is also possible that the region of lung tissue sampled may not be reflective of maturation-associated transcriptomic changes in other regions of the lung. Classical lung development studies by Brumley and colleagues suggested an asymmetric pattern of lung maturation, with a greater degree of maturation (as measured by pressure–volume characteristics) in the upper lobe as relative to the lower lobe of preterm lambs between 120 and 130 days’ gestation [68]. Accordingly, it is possible that these functionally distinct lobes also exert differential responses to ANS exposure, a topic presently under investigation by our group.

### Strengths and limitations

It is important to note that the data presented here were generated using a sheep model of pregnancy. Although a well validated system with which to assess fetal responses to ANS exposure in the early preterm period (i.e., approximating 32 weeks’ gestation), there are several limitations to using this system that should be considered when interpreting the data and its translational impact. Principal among these is the rigorous standardization of pregnancy and treatment (i.e., ANS regimen and ventilation protocol) variables. Although this allows us to isolate potential differences in ANS responsiveness to the fetus itself, such a scenario is a sizable departure from the heterogenous, highly variable use of ANS in a clinical setting. Although allowing us to select for dizygotic twins, the sheep model does not represent the full potential complexity of human twins (i.e., dizygotic vs. monozygotic and chorionic/amniotic variants therein). It is also important to note that the human and sheep placentas are structurally distinct, reflected in sizable differences in materno-fetal steroid gradients. How this difference impacts the relative involvement of fetal placental tissues vs. the fetus itself in determining lung maturation is unknown. Similarly, it is not clear how the potential inter-fetal transfer mediators such as sex hormones differs between humans and sheep. It should also be noted that the SNP associations identified in this study are not supported by functional data or an assessment of how these changes might impact their function. Lastly, this study was primarily undertaken to assess the concordance of ANS-induced lung maturation between twin pairs. We have reported a number of secondary findings of interest, in particular fetal sex as a potential modifier of ANS responsiveness, which should naturally be extrapolated with a reasonable degree of caution. Given the statistical model used (and the potential for collinearity effects), our result may be more reliable for mixed sex twin pairs, which represent the majority (65%) of our samples. Further analyses should be undertaken to specifically address this important observation, especially given recent findings of sex effects in the original ANS trial undertaken in New Zealand [39].

## Conclusions

In an analysis of preterm fetal sheep responses to ANS treatment, we show that twin ventilation outcomes and drug exposures are neither biologically nor statistically different to those of singleton fetuses. These data thus support the use of ANS in twin pregnancies, and similarly suggest that current ANS dosing for twins may be reduced. Our data also show, in a highly controlled experimental setting, that ANS-exposed twin male fetuses have poorer respiratory outcomes than female fetuses. Overall group analyses show that dizygotic twins had poor concordance in ANS responsiveness, with only 52% of twin pairs showing equivalence of ANS responsiveness. Based on these findings, and assuming no difference in input from the placental or maternal compartments, we conclude that fetal lung responses to ANS treatment in dizygotic twin preterm lambs are dependent on the fetus itself. Our data suggest the potential for a heritable role in determining ANS responsiveness in sheep. Glucocorticoid resistance is well established in several adult disease processes. Given this, and the data presented herein, further human studies investigating the potential for heritability to determine fetal ANS responsiveness are warranted. This is especially true considering an increased appreciation for the increasing use of ANS therapy and the potential neurodevelopmental risks associated with this standard of care.

## Supplementary Information

Additional File 1: Additional Tables 1–3, Additional Figs. 1–3. Additional Table 1. Twin zygosity, concordance values for twin sets. Additional Table 2. Fetal cortisol and adrenocorticotropic hormone (ACTH) levels at delivery by group. Additional Table 3. Differentially expressed messenger ribonucleic acid (mRNA) transcripts in the fetal lung, ANS responders vs. non-responders. Additional Fig. 1. Partial pressure of carbon dioxide (PaCO_2_) values recorded at 30 min of ventilation. Additional Fig. 2. Heatmap of Bulk RNA sequencing data stratified by treatment and ANS response status. Additional Fig. 3. Expression of mRNA transcript for surfactant proteins A-D measured by quantitative PCR.

## Data Availability

No datasets were generated or analysed during the current study.

## Abbreviations

ACTH: Adrenocorticotropic hormone
ALT: Alanine transaminase
ANOVA: Analysis of variance
ANS: Antenatal steroids
APIP: APAF1-interacting protein gene
AST: Aspartate aminotransferase
CAPN3: Calpain 3
CD53: CD53 molecule
CS1: Celestone 1 dose
CS2: Celestone 2 dose
IM: Intramuscular
DNA: Deoxyribonucleic acid
ECM: Extracellular matrix
EYA3: Eyes absent transcriptional coactivator and phosphatase 3
FDR: False discovery rate
GEE: Generalized estimation equation
GGT: Gamma-glutamyl transferase
GLDH: Glutamate dehydrogenase
GSEA: Gene set enrichment analysis
HGF: Hepatocyte growth factor
IDO1: Indoleamine 2,3-dioxygenase 1
IGF-1: Insulin-like growth factor 1
IGFBP-6: Insulin-like growth factor binding protein 6
IVH: Intraventricular hemorrhage
KEGG: Kyoto Encyclopedia of Genes and Genomes
LC-MS: Liquid Chromatography-Mass Spectrometry
LEPR: Leptin receptor
mRNA: Messenger ribonucleic acid
MET: MET proto-oncogene, receptor tyrosine kinase
MTBE: Methyl tert-butyl ether
NEC: Necrotizing enterocolitis
NR3C1: Nuclear receptor subfamily 3 group C member 1
OR: Odds ratio
PaCO_2_: Partial pressure of carbon dioxide
PaO_2_: Partial pressure of oxygen
PCA: Principal component analysis
PEEP: Positive end expiratory pressure
PIP: Peak inspiratory pressure
qPCR: Quantitative polymerase chain reaction
RDS: Respiratory distress syndrome
RIN: RNA Integrity Number
RNA: Ribonucleic acid
RT: Room temperature
SCD5: Stearoyl-CoA desaturase 5
SD: Standard deviation
SNP: Single-nucleotide polymorphism
SFTPA: Surfactant protein A
SFTPB: Surfactant protein B
SFTPC: Surfactant protein C
SFTPD: Surfactant protein D
SHROOM4: Shroom family member 4
STAT1: Signal transducer and activator of transcription 1
VEI: Ventilation efficacy index

## References

[1] Blencowe H, Cousens S, Chou D, et al. Born too soon: the global epidemiology of 15 million preterm births. Reprod Health. 2013;10 Suppl 1(Suppl 1):S2. 10.1186/1742-4755-10-S1-S2. 10.1186/1742-4755-10-S1-S2 PMC3828585 24625129

[2] Kemp MW, Saito M, Usuda H, Watanabe S, Sato S, Hanita T, et al. The efficacy of antenatal steroid therapy is dependent on the duration of low-concentration fetal exposure: evidence from a sheep model of pregnancy. Am J Obstet Gynecol. 2018;219(3):301.e1–e16. 29758177 10.1016/j.ajog.2018.05.007

[3] Ballard PL, Ballard RA. Scientific basis and therapeutic regimens for use of antenatal glucocorticoids. Am J Obstet Gynecol. 1995;173(1):254–62. 7631700 10.1016/0002-9378(95)90210-4

[4] McGoldrick E, Stewart F, Parker R, Dalziel SR. Antenatal corticosteroids for accelerating fetal lung maturation for women at risk of preterm birth. Cochrane Database Syst Rev. 2020;12(12):CD004454.10.1002/14651858.CD004454.pub4. 10.1002/14651858.CD004454.pub4 PMC8094626 33368142

[5] Jobe AH, Goldenberg RL. Antenatal corticosteroids: an assessment of anticipated benefits and potential risks. Am J Obstet Gynecol. 2018;219(1):62–74. 29630886 10.1016/j.ajog.2018.04.007

[6] Jobe AH, Goldenberg RL, Kemp MW. Antenatal corticosteroids: an updated assessment of anticipated benefits and potential risks. Am J Obstet Gynecol. 2024;230(3):330–9. 37734637 10.1016/j.ajog.2023.09.013

[7] Jobe AH, Kemp M, Schmidt A, Takahashi T, Newnham J, Milad M. Antenatal corticosteroids: a reappraisal of the drug formulation and dose. Pediatr Res. 2021;89(2):318–25. 33177675 10.1038/s41390-020-01249-w PMC7892336

[8] Takahashi T, Jobe AH, Fee EL, Newnham JP, Schmidt AF, Usuda H, et al. The complex challenge of antenatal steroid therapy nonresponsiveness. Am J Obstet Gynecol. 2022;227(5):696–704. 35932879 10.1016/j.ajog.2022.07.030

[9] Takahashi T, Takahashi Y, Fee EL, Saito M, Yaegashi N, Usuda H, et al. Continuous but not pulsed low-dose fetal betamethasone exposures extend the durability of antenatal steroid therapy. Am J Physiol Lung Cell Mol Physiol. 2022;322(6):L784–93. 35380907 10.1152/ajplung.00018.2022

[10] Takahashi T, Saito M, Schmidt AF, Usuda H, Takahashi Y, Watanabe S, et al. Variability in the efficacy of a standardized antenatal steroid treatment was independent of maternal or fetal plasma drug levels: evidence from a sheep model of pregnancy. Am J Obstet Gynecol. 2020;223(6):921.e1–921.e10. 32445634 10.1016/j.ajog.2020.05.032

[11] Kemp MW, Saito M, Schmidt AF, Usuda H, Watanabe S, Sato S, et al. The duration of fetal antenatal steroid exposure determines the durability of preterm ovine lung maturation. Am J Obstet Gynecol. 2020;222(2):183.e1-.e9. 31494126 10.1016/j.ajog.2019.08.046

[12] Rowson LE, Moor R. Occurrence and development of identical twins in sheep. Nature. 1964;201:521–2. 14164644 10.1038/201521a0

[13] Faul F, Erdfelder E, Lang A-G, Buchner A. G*Power 3: a flexible statistical power analysis program for the social, behavioral, and biomedical sciences. Behav Res Methods. 2007;39(2):175–91. 17695343 10.3758/bf03193146

[14] Takahashi T, Fee EL, Takahashi Y, Saito M, Yaegashi N, Usuda H, et al. Betamethasone phosphate reduces the efficacy of antenatal steroid therapy and is associated with lower birth weights when administered to pregnant sheep in combination with betamethasone acetate. Am J Obstet Gynecol. 2021. 10.1016/j.ajog.2021.10.001 34626553

[15] Notter RH, Egan EA, Kwong MS, Holm BA, Shapiro DL. Lung surfactant replacement in premature lambs with extracted lipids from bovine lung lavage: effects of dose, dispersion technique, and gestational age. Pediatr Res. 1985;19(6):569–77. 3839302 10.1203/00006450-198506000-00014

[16] Ewels PA, Peltzer A, Fillinger S, Patel H, Alneberg J, Wilm A, et al. The nf-core framework for community-curated bioinformatics pipelines. Nat Biotechnol. 2020;38(3):276–8. 32055031 10.1038/s41587-020-0439-x

[17] Di Tommaso P, Chatzou M, Floden EW, Barja PP, Palumbo E, Notredame C. Nextflow enables reproducible computational workflows. Nat Biotechnol. 2017;35(4):316–9. 28398311 10.1038/nbt.3820

[18] Krueger F. Trim Galore!: A wrapper around Cutadapt and FastQC to consistently apply adapter and quality trimming to FastQ files, with extra functionality for RRBS data. Babraham Institute; 2015. https://scholar.google.com/scholar_lookup?title=Trim+galore%3A+a+wrapper+around+cutadapt+and+fastqc+to+consistently+apply+adapter+and+quality+trimming+to+fastq+files%2C+with+extra+functionality+for+Rr.

[19] Dobin A, Davis CA, Schlesinger F, Drenkow J, Zaleski C, Jha S, et al. STAR: ultrafast universal RNA-seq aligner. Bioinformatics. 2013;29(1):15–21. 23104886 10.1093/bioinformatics/bts635 PMC3530905

[20] Patro R, Duggal G, Love MI, Irizarry RA, Kingsford C. Salmon provides fast and bias-aware quantification of transcript expression. Nat Methods. 2017;14(4):417–9. 28263959 10.1038/nmeth.4197 PMC5600148

[21] R Core Team. R: A Language and Environment for Statistical Computing.Vienna: R Foundation for Statistical Computing; 2013.

[22] Love MI, Huber W, Anders S. Moderated estimation of fold change and dispersion for RNA-seq data with DESeq2. Genome Biol. 2014;15(12):1–21. 10.1186/s13059-014-0550-8 PMC4302049 25516281

[23] Blighe K, Rana S, Lewis M. EnhancedVolcano: publication-ready volcano plots with enhanced colouring and labeling. R package version. 2019;1(0):10–8129.

[24] Gu Z, Eils R, Schlesner M. Complex heatmaps reveal patterns and correlations in multidimensional genomic data. Bioinformatics. 2016;32(18):2847–9. 27207943 10.1093/bioinformatics/btw313

[25] Wu T, Hu E, Xu S, Chen M, Guo P, Dai Z, et al. ClusterProfiler 4.0: a universal enrichment tool for interpreting omics data. The innovation. 2021;2(3):100141. 34557778 10.1016/j.xinn.2021.100141 PMC8454663

[26] Kanehisa M, Goto S. KEGG: kyoto encyclopedia of genes and genomes. Nucleic Acids Res. 2000;28(1):27–30. 10592173 10.1093/nar/28.1.27 PMC102409

[27] Kemp MW, Saito M, Usuda H, Molloy TJ, Miura Y, Sato S, et al. Maternofetal pharmacokinetics and fetal lung responses in chronically catheterized sheep receiving constant, low-dose infusions of betamethasone phosphate. Am J Obstet Gynecol. 2016;215(6):775.e1. 27555319 10.1016/j.ajog.2016.08.017

[28] Melamed N, Shah J, Yoon EW, Pelausa E, Lee SK, Shah PS, et al. The role of antenatal corticosteroids in twin pregnancies complicated by preterm birth. Am J Obstet Gynecol. 2016;215(4):482.e1-4829. 27260974 10.1016/j.ajog.2016.05.037

[29] Vaz A, Malheiro MF, Severo M, Rodrigues T, Guimarães H, Montenegro N. Effect of antenatal corticosteroids on morbidity and mortality of preterm singletons and twins. J Matern Fetal Neonatal Med. 2018;31(6):754–60. 28277916 10.1080/14767058.2017.1297408

[30] Palas D, Ehlinger V, Alberge C, Truffert P, Kayem G, Goffinet F, et al. Efficacy of antenatal corticosteroids in preterm twins: the EPIPAGE-2 cohort study. BJOG. 2018;125(9):1164–70. 29119673 10.1111/1471-0528.15014

[31] Ben-David A, Zlatkin R, Bookstein-Peretz S, Meyer R, Mazaki-Tovi S, Yinon Y. Does antenatal steroids treatment in twin pregnancies prior to late preterm birth reduce neonatal morbidity? Evidence from a retrospective cohort study. Arch Gynecol Obstet. 2020;302(5):1121–6. 32728923 10.1007/s00404-020-05709-w

[32] Zhu J, Zhao Y, An P, Zhao Y, Li S, Zhou J, et al. Antenatal corticosteroid treatment during the late-preterm period and neonatal outcomes for twin pregnancies. JAMA Network Open. 2023;6(11):e2343781-e. 37976061 10.1001/jamanetworkopen.2023.43781 PMC10656637

[33] Hong S, Lee SM, Kwak DW, Lee J, Kim SY, Oh JW, et al. Effects of antenatal corticosteroids in twin neonates with late preterm birth (ACTWIN [Antenatal Corticosteroids in TWIN late preterm neonates] trial): study protocol for a randomized controlled trial. BMC Pregnancy Childbirth. 2019;19(1):114. 30943910 10.1186/s12884-019-2235-5 PMC6446272

[34] Murray S, Thompson J, Townsend RC, Deidda M, Boyd KA, Norman JE, et al. Randomised placebo-controlled trial of antenatal corticosteroids for planned birth in twins (STOPPIT-3): study protocol. BMJ Open. 2024;14(1):e078778. 38238048 10.1136/bmjopen-2023-078778 PMC10806667

[35] Kramer BW. Chorioamnionitis - new ideas from experimental models. Neonatology. 2011;99(4):320–5. 21701204 10.1159/000326620

[36] Ikegami M, Jobe AH, Newnham J, Polk DH, Willet KE, Sly P. Repetitive prenatal glucocorticoids improve lung function and decrease growth in preterm lambs. American Journal of Respiratory and Critical Care Medicine. 1997;156(1):178–84. 9230744 10.1164/ajrccm.156.1.9612036

[37] Ballard PL, Ballard RA, Granberg JP, Sniderman S, Gluckman PD, Kaplan SL, et al. Fetal sex and prenatal betamethasone therapy. J Pediatr. 1980;97(3):451–4. 7411310 10.1016/s0022-3476(80)80204-6

[38] Ramos-Navarro C, Sánchez-Luna M, Zeballos-Sarrato S, Pescador-Chamorro I. Antenatal corticosteroids and the influence of sex on morbidity and mortality of preterm infants. J Matern Fetal Neonatal Med. 2022;35(18):3438–45. 32933373 10.1080/14767058.2020.1819977

[39] Walters AGB, Lin L, Crowther CA, Gamble GD, Dalziel SR, Harding JE. Betamethasone for preterm birth: Auckland Steroid Trial full results and new insights 50 years on. J Pediatr. 2023;255:80-8.e5. 36336005 10.1016/j.jpeds.2022.10.028

[40] Roberge S, Lacasse Y, Tapp S, Tremblay Y, Kari A, Liu J, et al. Role of fetal sex in the outcome of antenatal glucocorticoid treatment to prevent respiratory distress syndrome: systematic review and meta-analysis. J Obstet Gynaecol Can. 2011;33(3):216–26. 21453561 10.1016/s1701-2163(16)34822-8

[41] Della Torre M, Hibbard JU, Jeong H, Fischer JH. Betamethasone in pregnancy: influence of maternal body weight and multiple gestation on pharmacokinetics. Am J Obstet Gynecol. 2010;203(3):254.e1-2512. 20816148 10.1016/j.ajog.2010.06.029 PMC4326076

[42] Ballabh P, Lo ES, Kumari J, Cooper TB, Zervoudakis I, Auld PAM, et al. Pharmacokinetics of betamethasone in twin and singleton pregnancy. Clin Pharmacol Ther. 2002;71(1):39–45. 11823756 10.1067/mcp.2002.120250

[43] Roman A, Ramirez A, Fox NS. Prevention of preterm birth in twin pregnancies. Am J Obstet Gynecol MFM. 2022;4(2s):100551. 34896357 10.1016/j.ajogmf.2021.100551

[44] Refuerzo JS, Momirova V, Peaceman AM, Sciscione A, Rouse DJ, Caritis SN, et al. Neonatal outcomes in twin pregnancies delivered moderately preterm, late preterm, and term. Am J Perinatol. 2010;27(7):537–42. 20175042 10.1055/s-0030-1248940 PMC2990398

[45] van Rossum EF, Koper JW, van den Beld AW, Uitterlinden AG, Arp P, Ester W, et al. Identification of the BclI polymorphism in the glucocorticoid receptor gene: association with sensitivity to glucocorticoids in vivo and body mass index. Clin Endocrinol (Oxf). 2003;59(5):585–92. 14616881 10.1046/j.1365-2265.2003.01888.x

[46] Haas DM, Lehmann AS, Skaar T, Philips S, McCormick CL, Beagle K, et al. The impact of drug metabolizing enzyme polymorphisms on outcomes after antenatal corticosteroid use. Am J Obstet Gynecol. 2012;206(5):447.e17-44724. 22445700 10.1016/j.ajog.2012.02.016 PMC3340461

[47] Panek M, Pietras T, Antczak A, Gorski P, Kuna P, Szemraj J. The role of functional single nucleotide polymorphisms of the human glucocorticoid receptor gene NR3C1 in Polish patients with bronchial asthma. Mol Biol Rep. 2012;39(4):4749–57. 22015776 10.1007/s11033-011-1267-3 PMC3294211

[48] Liso A, Venuto S, Coda ARD, Giallongo C, Palumbo GA, Tibullo D. IGFBP-6: at the crossroads of immunity, tissue repair and fibrosis. Int J Mol Sci. 2022;23(8):4358. 35457175 10.3390/ijms23084358 PMC9030159

[49] van de Wetering JK, Elfring RH, Oosterlaken-Dijksterhuis MA, Mol JA, Haagsman HP, Batenburg JJ. Perinatal expression of IGFBPs in rat lung and its hormonal regulation in fetal lung explants. Am J Physiol. 1997;273(6):L1174–81. 9435572 10.1152/ajplung.1997.273.6.L1174

[50] Fang X, Liu S, Cheng Y, Li S, Wu Q, Su D, et al. SNPs in the 5’ terminal-region of IGFBP6 gene and its linkage with pig body size. Animal Cells and Systems. 2015;19(6):417–24.

[51] Wang Y, Pandey RN, York AJ, Mallela J, Nichols WC, Hu Y-C, et al. The EYA3 tyrosine phosphatase activity promotes pulmonary vascular remodeling in pulmonary arterial hypertension. Nat Commun. 2019;10(1):4143. 31515519 10.1038/s41467-019-12226-1 PMC6742632

[52] Liu X, Wang M, Jiang T, He J, Fu X, Xu Y. IDO1 maintains pluripotency of primed human embryonic stem cells by promoting glycolysis. Stem Cells. 2019;37(9):1158–65. 31145821 10.1002/stem.3044

[53] Maeda Y, Davé V, Whitsett JA. Transcriptional control of lung morphogenesis. Physiol Rev. 2007;87(1):219–44. 17237346 10.1152/physrev.00028.2006

[54] Aros CJ, Pantoja CJ, Gomperts BN. Wnt signaling in lung development, regeneration, and disease progression. Communications Biology. 2021;4(1):601. 34017045 10.1038/s42003-021-02118-w PMC8138018

[55] Zámbó V, Orosz G, Szabó L, Tibori K, Sipeki S, Molnár K, et al. A single nucleotide polymorphism (rs3811792) affecting human SCD5 promoter activity is associated with diabetes mellitus. Genes. 2022;13(10):1784. 36292669 10.3390/genes13101784 PMC9601412

[56] Organ SL, Tsao MS. An overview of the c-MET signaling pathway. Ther Adv Med Oncol. 2011;3(1 Suppl):S7-s19. 22128289 10.1177/1758834011422556 PMC3225017

[57] Ware LB, Matthay MA. Keratinocyte and hepatocyte growth factors in the lung: roles in lung development, inflammation, and repair. American Journal of Physiology-Lung Cellular and Molecular Physiology. 2002;282(5):L924–40. 11943656 10.1152/ajplung.00439.2001

[58] Panganiban RA, Day RM. Hepatocyte growth factor in lung repair and pulmonary fibrosis. Acta Pharmacol Sin. 2011;32(1):12–20. 21131996 10.1038/aps.2010.90 PMC4003323

[59] Fu J, Su X, Li Z, Deng L, Liu X, Feng X, et al. HGF/c-MET pathway in cancer: from molecular characterization to clinical evidence. Oncogene. 2021;40(28):4625–51. 34145400 10.1038/s41388-021-01863-w

[60] Severgnini M, Takahashi S, Rozo LM, Homer RJ, Kuhn C, Jhung JW, et al. Activation of the STAT pathway in acute lung injury. American Journal of Physiology-Lung Cellular and Molecular Physiology. 2004;286(6):L1282–92. 14729509 10.1152/ajplung.00349.2003

[61] Tolomeo M, Cavalli A, Cascio A. STAT1 and its crucial role in the control of viral infections. Int J Mol Sci. 2022;23(8):4095. 35456913 10.3390/ijms23084095 PMC9028532

[62] Xu Y, Wang Y, Besnard V, Ikegami M, Wert SE, Heffner C, et al. Transcriptional programs controlling perinatal lung maturation. PLoS One. 2012;7(8):e37046. 22916088 10.1371/journal.pone.0037046 PMC3423373

[63] Laube M, Dornis D, Wenzel F, Thome UH. Epidermal growth factor strongly affects epithelial Na(+) transport and barrier function in fetal alveolar cells, with minor sex-specific effects. Sci Rep. 2021;11(1):15951. 34354180 10.1038/s41598-021-95410-y PMC8342687

[64] Mulder EJH, Derks JB, Visser GHA. Effects of antenatal betamethasone administration on fetal heart rate and behavior in twin pregnancy. Pediatr Res. 2004;56(1):35–9. 15128914 10.1203/01.PDR.0000130476.97700.2B

[65] Luke B, Hediger M, Min S-J, Brown MB, Misiunas RB, Gonzalez-Quintero VH, et al. Gender mix in twins and fetal growth, length of gestation and adult cancer risk. Paediatr Perinat Epidemiol. 2005;19(s1):41–7. 15670121 10.1111/j.1365-3016.2005.00616.x

[66] Lummaa V, Pettay JE, Russell AF. Male twins reduce fitness of female co-twins in humans. Proc Natl Acad Sci U S A. 2007;104(26):10915–20. 17576931 10.1073/pnas.0605875104 PMC1904168

[67] Bridges JP, Sudha P, Lipps D, Wagner A, Guo M, Du Y, et al. Glucocorticoid regulates mesenchymal cell differentiation required for perinatal lung morphogenesis and function. Am J Physiol Lung Cell Mol Physiol. 2020;319(2):L239–55. 32460513 10.1152/ajplung.00459.2019 PMC7473939

[68] Brumley GW, Chernick V, Hodson WA, Normand C, Fenner A, Avery ME. Correlations of mechanical stability, morphology, pulmonary surfactant, and phospholipid content in the developing lamb lung. J Clin Invest. 1967;46(5):863–73. 6025487 10.1172/JCI105586 PMC297088

