# Supplementary material for: Single-nucleotide polymorphisms in dizygotic twin ovine fetuses are associated with discordant responses to antenatal steroid therapy
Source: BMC Med. 2025 Feb 4;23:65. doi: 10.1186/s12916-025-03910-9 (PMC11792249; doi:10.1186/s12916-025-03910-9)
Supplement: Supplementary file 1 — Additional File 1: Additional Tables 1–3, Additional Figs. 1–3. Additional Table 1. Twin zygosity, concordance values for twin sets. Additional Table 2. Fetal cortisol and adrenocorticotropic hormone (ACTH) levels at delivery by group. Additional Table 3. Differentially expressed messenger ribonucleic acid (mRNA) transcripts in the fetal lung, ANS responders vs. non-responders. Additional Fig. 1. Partial pressure of carbon dioxide (PaCO2) values recorded at 30 min of ventilation. Additional Fig. 2. Heatmap of Bulk RNA sequencing data stratified by treatment and ANS response status. Additional Fig. 3. Expression of mRNA transcript for surfactant proteins A-D measured by quantitative PCR. [file 12916_2025_3910_MOESM1_ESM.docx]

**[ADDITIONAL FILE 1]**

Additional Table 1. Twin zygosity, concordance values for twin sets.

| **ADDITIONAL TABLE 1**  **Twin Zygosity, Concordance Values for Twin Sets.** | | | |
| --- | --- | --- | --- |
| **Animal ID** | **Twin Set** | **Concordance Rate** | **Zygosity** |
| 21.250 | 1 | 0.75 | Dizygotic |
| 21.251 | 1 |  |  |
| 21.252 | 2 | 0.76 | Dizygotic |
| 21.253 | 2 |  |  |
| 21.254 | 3 | 0.75 | Dizygotic |
| 21.255 | 3 |  |  |
| 21.256 | 4 | 0.74 | Dizygotic |
| 21.257 | 4 |  |  |
| 21.258 | 5 | 0.74 | Dizygotic |
| 21.259 | 5 |  |  |
| 21.260 | 6 | 0.75 | Dizygotic |
| 21.261 | 6 |  |  |
| 21.262 | 7 | 0.80 | Dizygotic |
| 21.263 | 7 |  |  |
| 21.264 | 8 | 0.75 | Dizygotic |
| 21.265 | 8 |  |  |
| 21.266 | 9 | 0.74 | Dizygotic |
| 21.267 | 9 |  |  |
| 21.268 | 10 | 0.77 | Dizygotic |
| 21.269 | 10 |  |  |
| 21.270 | 11 | 0.82 | Dizygotic |
| 21.271 | 11 |  |  |
| 21.272 | 12 | 0.82 | Dizygotic |
| 21.273 | 12 |  |  |
| 21.274 | 13 | 0.73 | Dizygotic |
| 21.275 | 13 |  |  |
| 21.276 | 14 | 0.76 | Dizygotic |
| 21.277 | 14 |  |  |
| 21.278 | 15 | 0.75 | Dizygotic |
| 21.279 | 15 |  |  |
| 21.280 | 16 | 0.75 | Dizygotic |
| 21.281 | 16 |  |  |
| 21.282 | 17 | 0.81 | Dizygotic |
| 21.283 | 17 |  |  |
| 21.284 | 18 | 0.74 | Dizygotic |
| 21.285 | 18 |  |  |
| 21.286 | 19 | 0.83 | Dizygotic |
| 21.287 | 19 |  |  |
| 21.288 | 20 | 0.80 | Dizygotic |
| 21.289 | 20 |  |  |
| 21.290 | 21 | 0.74 | Dizygotic |
| 21.291 | 21 |  |  |
| 21.292 | 22 | 0.74 | Dizygotic |
| 21.293 | 22 |  |  |
| 21.294 | 23 | 0.81 | Dizygotic |
| 21.295 | 23 |  |  |
| 21.296 | 24 | 0.76 | Dizygotic |
| 21.297 | 24 |  |  |
| 21.298 | 25 | 0.75 | Dizygotic |
| 21.299 | 25 |  |  |
| 21.300 | 26 | 0.81 | Dizygotic |
| 21.301 | 26 |  |  |
| 21.302 | 27 | 0.76 | Dizygotic |
| 21.303 | 27 |  |  |
| 21.304 | 28 | 0.73 | Dizygotic |
| 21.305 | 28 |  |  |
| 21.308 | 29 | 0.81 | Dizygotic |
| 21.309 | 29 |  |  |
| 21.310 | 30 | 0.75 | Dizygotic |
| 21.311 | 30 |  |  |
| 21.312 | 31 | 0.74 | Dizygotic |
| 21.313 | 31 |  |  |

**Additional Table 2. Fetal cortisol and adrenocorticotropic hormone (ACTH) levels at delivery by group.**

| **ADDITIONAL TABLE 2**  **Fetal Cortisol and ACTH Levels at Delivery by Group.** | | |
| --- | --- | --- |
| **Treatment Group** | **Cortisol (nmol / L)** | **ACTH (pg / mL)** |
| CS1 | <5.5 | 10 |
| CS1 | <5.5 | 15 |
| CS1 | <5.5 | 5 |
| CS1 | <5.5 | 8 |
| CS1 | <5.5 | 10 |
| CS1 | <5.5 | 12 |
| CS1 | <5.5 | 9 |
| CS1 | <5.5 | 10 |
| CS1 | <5.5 | 12 |
| CS1 | <5.5 | 14 |
| CS1 | <5.5 | 7 |
| CS1 | <5.5 | 10 |
| CS1 | <5.5 | 5.6 |
| CS1 | <5.5 | <5 |
| CS1 | <5.5 | 7.21 |
| CS1 | <5.5 | 8.83 |
| CS1 | <5.5 | 5.92 |
| CS1 | <5.5 | 7.52 |
| CS1 | <5.5 | <5 |
| CS1 | <5.5 | <5 |
| CS1 | <5.5 | <5 |
| CS1 | <5.5 | 5.56 |
| CS2 | <5.5 | 7.8 |
| CS2 | <5.5 | 7.9 |
| CS2 | <5.5 | 9 |
| CS2 | <5.5 | 12 |
| CS2 | <5.5 | 6 |
| CS2 | <5.5 | 6.1 |
| CS2 | <5.5 | 6.82 |
| CS2 | <5.5 | 8.22 |
| CS2 | <5.5 | <5.5 |
| CS2 | <5.5 | 7.64 |
| CS2 | <5.5 | <5 |
| CS2 | <5.5 | 8.17 |
| CS2 | * | * |
| CS2 | <5.5 | 13.7 |
| CS2 | <5.5 | 21.7 |
| CS2 | <5.5 | 11.2 |
| CS2 | * | * |
| CS2 | * | * |
| CS2 | <5.5 | 5.81 |
| CS2 | <5.5 | 7.91 |
| Negative Control Group | 17.1 | 324 |
| Negative Control Group | 13.7 | 420 |
| Negative Control Group | 19.4 | 643 |
| Negative Control Group | 30.1 | 589 |
| Negative Control Group | 10.4 | 564 |
| Negative Control Group | 19.5 | 657 |
| Negative Control Group | 15.9 | 926 |
| Negative Control Group | 17.4 | 459 |
| Negative Control Group | 6.87 | >1250 |
| Negative Control Group | 7.89 | >1250 |
| Negative Control Group | 8.66 | >1250 |
| Negative Control Group | 18.6 | >1250 |
| Negative Control Group | 11.3 | 255 |
| Negative Control Group | 27.9 | >1250 |
| Negative Control Group | 15.4 | 1159 |
| Negative Control Group | 11.8 | >1250 |
| Negative Control Group | 11.6 | >1250 |
| Negative Control Group | 16.1 | >1250 |
| Negative Control Group | 19.2 | >1250 |
| Negative Control Group | 17.2 | * |
| **Missing data*  *Limit of detection for blood chemistry assays: Cortisol lower limit 5.5 (nmol / L), ACTH lower limit 5 pg / mL, upper limit 1250 pg / mL* | | |

**Additional Table 3. Differentially expressed messenger ribonucleic acid (mRNA) transcripts in the fetal lung, ANS responders vs. non-responders.**

| **ADDITIONAL TABLE 3**  **Differentially Expressed RNA Transcripts in the Fetal Lung, ANS Responders (n=24) vs. Non-Responders (n=18).** | | | | | | |
| --- | --- | --- | --- | --- | --- | --- |
| **SYMBOL** | **ENTREZID** | **GENENAME** | **baseMean** | **Fold Change** | **p** | **FDR** |
| **Up Regulated Genes** | | | | | | |
| LOC101117184 | 101117184 | metallothionein-1A | 11 | 3.76 | 2.5E-06 | 1.9E-03 |
| RHEX | 101111896 | regulator of hemoglobinization and erythroid cell expansion | 16 | 2.25 | 2.5E-05 | 9.1E-03 |
| ARID5A | 101114695 | AT-rich interaction domain 5A | 563 | 2.23 | 1.3E-04 | 2.5E-02 |
| RNF122 | 101116204 | ring finger protein 122 | 272 | 1.92 | 2.1E-04 | 3.3E-02 |
| ACTRT3 | 101120792 | actin related protein T3 | 35 | 1.78 | 2.2E-04 | 3.3E-02 |
| SLC16A6 | 101116069 | solute carrier family 16 member 6 | 673 | 1.65 | 3.1E-04 | 4.1E-02 |
| TNFRSF12A | 114110580 | TNF receptor superfamily member 12A | 463 | 1.59 | 4.1E-04 | 4.7E-02 |
| SPAG4 | 101120855 | sperm associated antigen 4 | 48 | 1.55 | 1.0E-04 | 2.3E-02 |
| LOC114110632 | 114110632 | uncharacterized LOC114110632 | 50 | 1.52 | 3.7E-04 | 4.5E-02 |
| **Down Regulated Genes** | | | | | | |
| LOC101123292 | 101123292 | histone H2A type 3 | 88 | -1.54 | 1.6E-05 | 7.4E-03 |
| ALDH1L1 | 100913154 | aldehyde dehydrogenase 1 family member L1 | 56 | -1.64 | 1.1E-04 | 2.4E-02 |
| KCNH5 | 101106850 | potassium voltage-gated channel subfamily H member 5 | 65 | -1.66 | 1.0E-04 | 2.4E-02 |
| DEPP1 | 101120888 | DEPP autophagy regulator 1 | 1810 | -1.73 | 4.2E-06 | 2.6E-03 |
| LOC105603814 | 105603814 | histone H3.1 | 42 | -1.84 | 3.8E-04 | 4.6E-02 |
| LOC105610456 | 105610456 | glutaredoxin-related protein 5, mitochondrial-like | 68 | -1.88 | 2.1E-07 | 3.8E-04 |
| LOC114110111 | 114110111 | uncharacterized LOC114110111 | 22 | -1.91 | 1.7E-05 | 7.4E-03 |
| LOC101105036 | 101105036 | histone H2A type 1 | 31 | -2.01 | 2.7E-05 | 9.3E-03 |
| LOC121817641 | 121817641 | uncharacterized LOC121817641 | 23 | -2.31 | 2.0E-04 | 3.2E-02 |
| LOC101113971 | 101113971 | RUN and FYVE domain-containing protein 1-like | 15 | -3.20 | 3.4E-04 | 4.3E-02 |
| LOC101108033 | 101108033 | 60S ribosomal protein L18a-like | 53 | -8.17 | 1.2E-04 | 2.5E-02 |
| LOC101123533 | 101123533 | 60S ribosomal protein L37a | 91 | -17.75 | 2.4E-13 | 1.3E-09 |
| LOC101121371 | 101121371 | 60S ribosomal protein L37a | 443 | -18.51 | 6.3E-09 | 2.6E-05 |
| LOC114109518 | 114109518 | 60S ribosomal protein L37a | 210 | -22.47 | 7.6E-15 | 1.2E-10 |

**

**

**Additional Figure 1. Partial pressure of carbon dioxide (PaCO_2_) values recorded at 30 minutes of ventilation.** *CS, Celestone.*

**
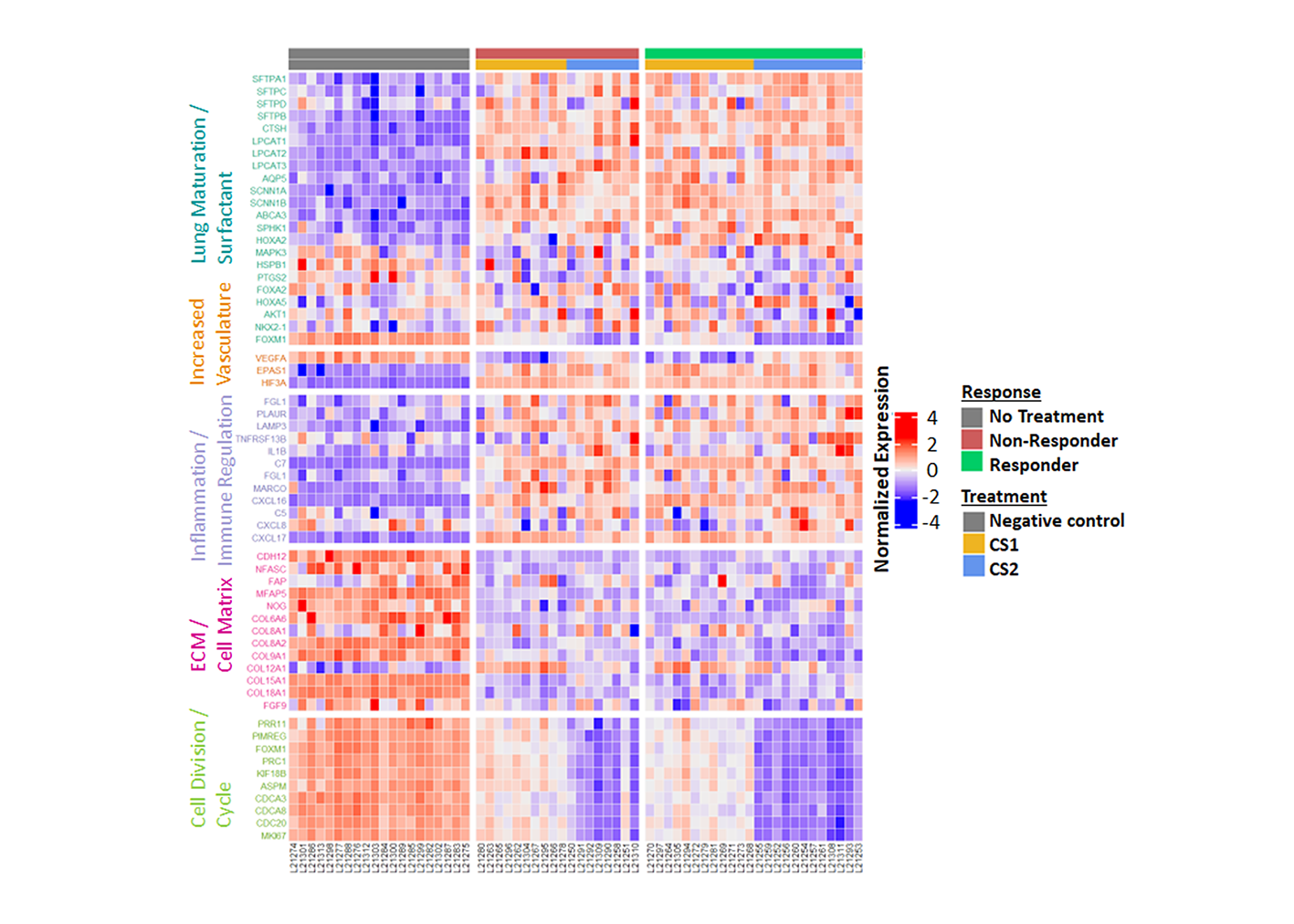
Additional Figure 2. Heatmap of Bulk RNA sequencing data stratified by treatment and ANS response status.**


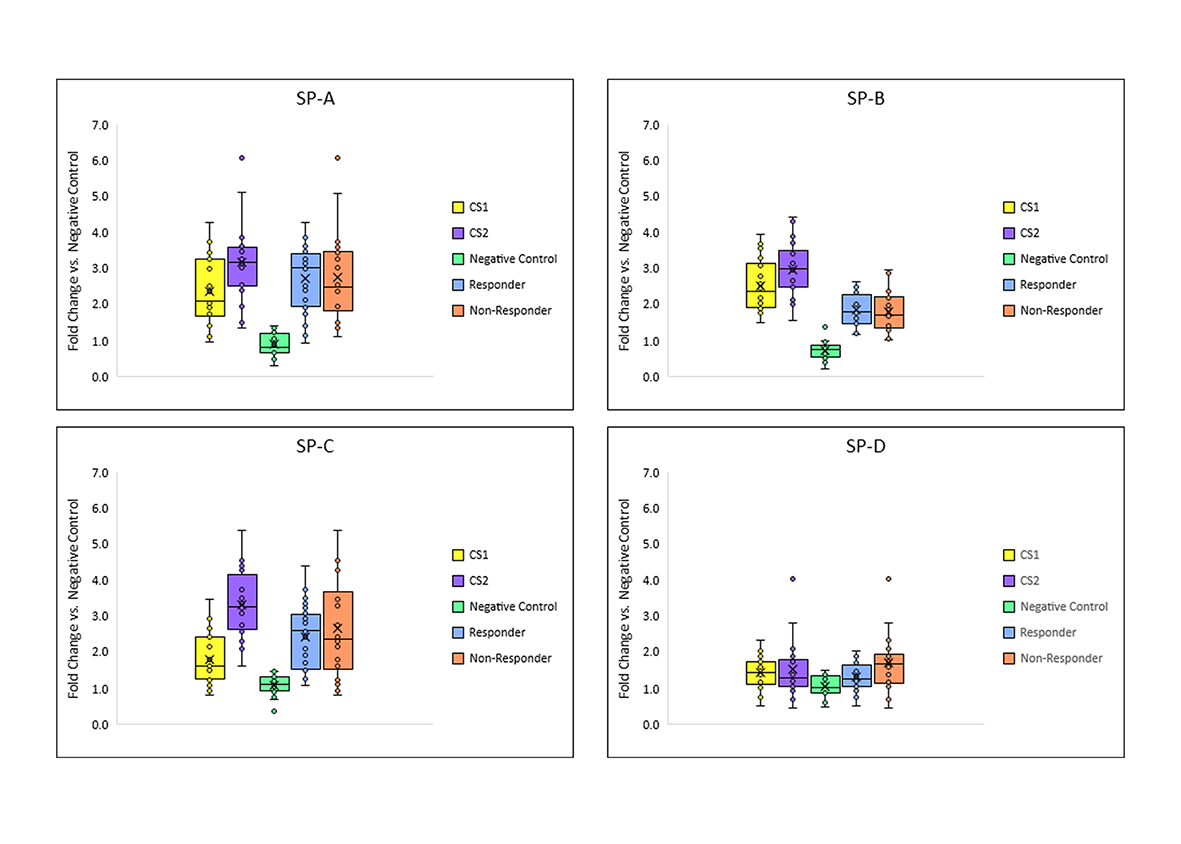
**Additional Figure 3. Expression of mRNA transcript for surfactant proteins A-D measured by quantitative PCR.**

*SFTPA, surfactant protein A; SFTPB, surfactant protein B; SFTPC, surfactant protein C; SFTPD, surfactant protein D.*
